# Supplementary material for: Expression of GLOD4 in the Testis of the Qianbei Ma Goat and Its Effect on Leydig Cells
Source: Animals (Basel). 2024 Sep 8;14(17):2611. doi: 10.3390/ani14172611 (PMC11393997; doi:10.3390/ani14172611)
Supplement: Supplementary file 1 [file animals-14-02611-s001.zip › flow cytometry images/flow cytometry-pcNDNA3.1-GLOD4.pptx]

## Slide 1
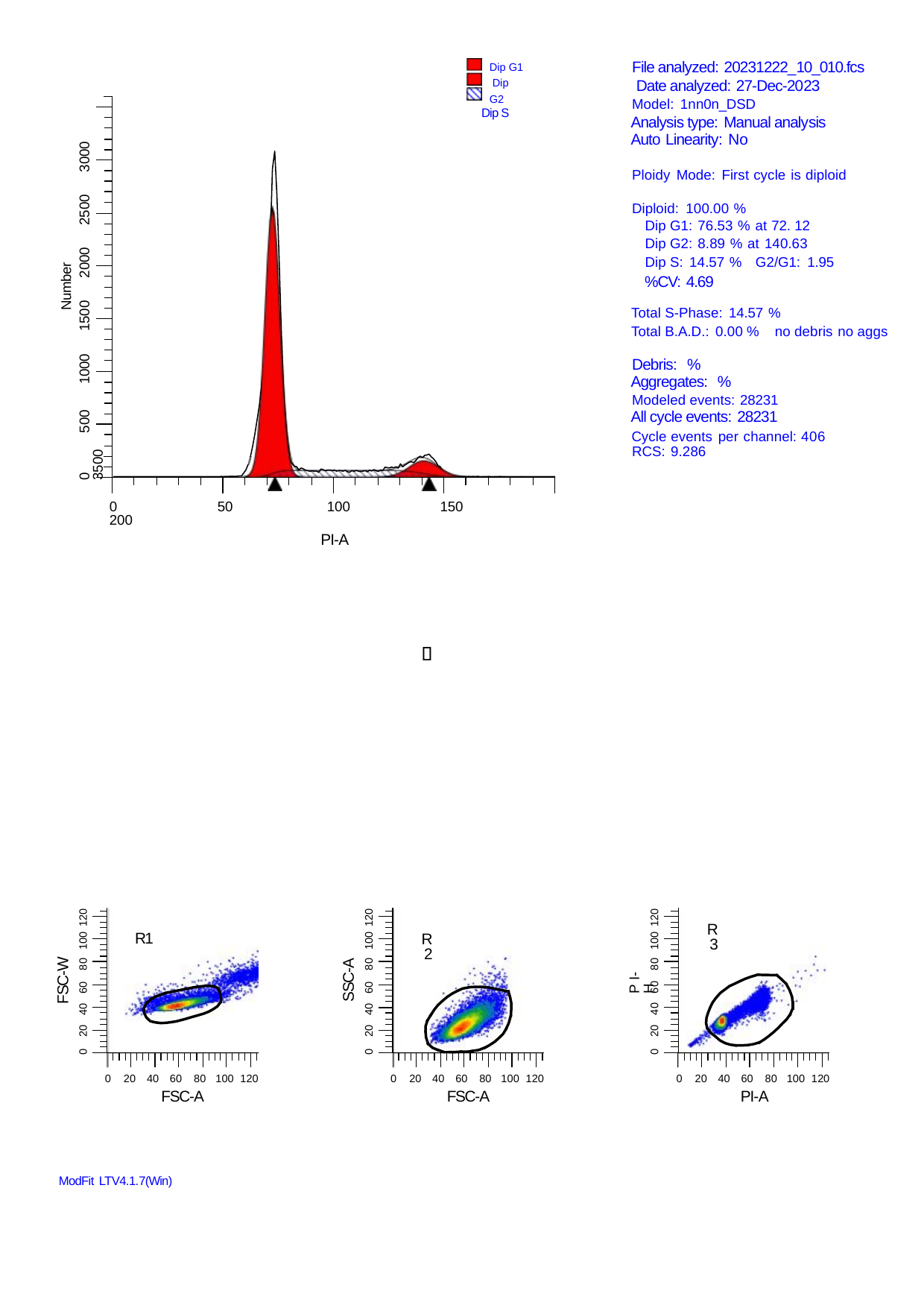

Dip G1 Dip G2
File analyzed: 20231222_10_010.fcs Date analyzed: 27-Dec-2023
Model: 1nn0n_DSD
Analysis type: Manual analysis
Auto Linearity: No
Ploidy Mode: First cycle is diploid
Diploid: 100.00 %
Dip G1: 76.53 % at 72. 12
Dip G2: 8.89 % at 140.63
Dip S: 14.57 % G2/G1: 1.95
%CV: 4.69
Total S-Phase: 14.57 %
Total B.A.D.: 0.00 % no debris no aggs
Debris: %
Aggregates: %
Modeled events: 28231
All cycle events: 28231
Cycle events per channel: 406
RCS: 9.286
	Dip S
Number
0 500 1000 1500 2000 2500 3000 3500
0 50 100 150 200
PI-A
| 用 |
| --- |
| 用 |
| --- |
| 用 |
| --- |
| 用 |
| --- |
R3
R1
R2
P I-H
SSC-A
FSC-W
0 20 40 60 80 100 120
0 20 40 60 80 100 120
0 20 40 60 80 100 120
0 20 40 60 80 100 120
FSC-A
0 20 40 60 80 100 120
FSC-A
0 20 40 60 80 100 120
PI-A
ModFit LTV4.1.7(Win)

## Slide 2
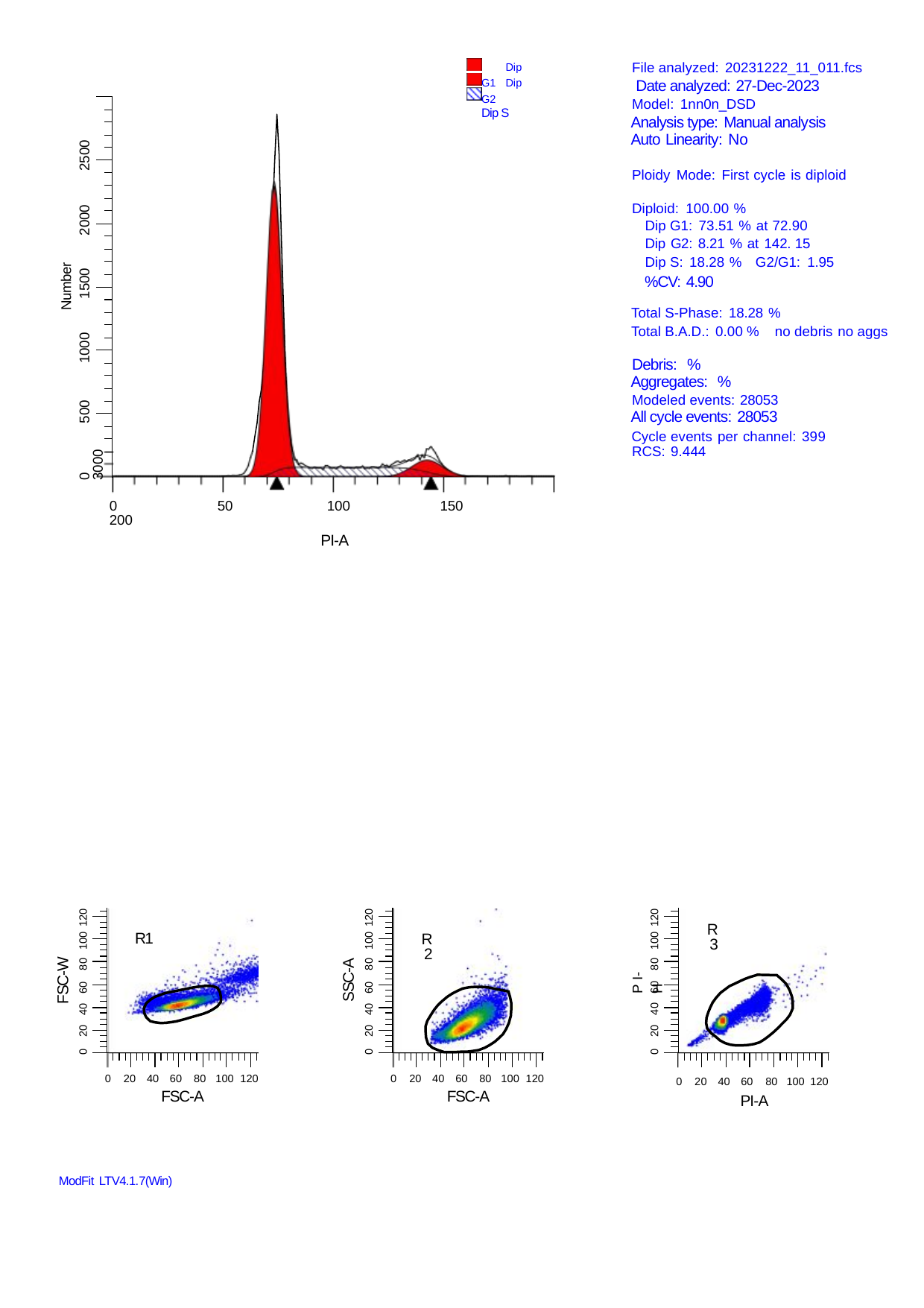

Dip G1 	Dip G2
File analyzed: 20231222_11_011.fcs Date analyzed: 27-Dec-2023
Model: 1nn0n_DSD
Analysis type: Manual analysis
Auto Linearity: No
Ploidy Mode: First cycle is diploid
Diploid: 100.00 %
Dip G1: 73.51 % at 72.90
Dip G2: 8.21 % at 142. 15
Dip S: 18.28 % G2/G1: 1.95
%CV: 4.90
Total S-Phase: 18.28 %
Total B.A.D.: 0.00 % no debris no aggs
Debris: %
Aggregates: %
Modeled events: 28053
All cycle events: 28053
Cycle events per channel: 399
RCS: 9.444
	Dip S
0 500 1000 1500 2000 2500 3000
Number
0 50 100 150 200
PI-A
R3
R1
R2
P I-H
SSC-A
FSC-W
0 20 40 60 80 100 120
0 20 40 60 80 100 120
0 20 40 60 80 100 120
0 20 40 60 80 100 120
FSC-A
0 20 40 60 80 100 120
FSC-A
0 20 40 60 80 100 120 PI-A
ModFit LTV4.1.7(Win)

## Slide 3
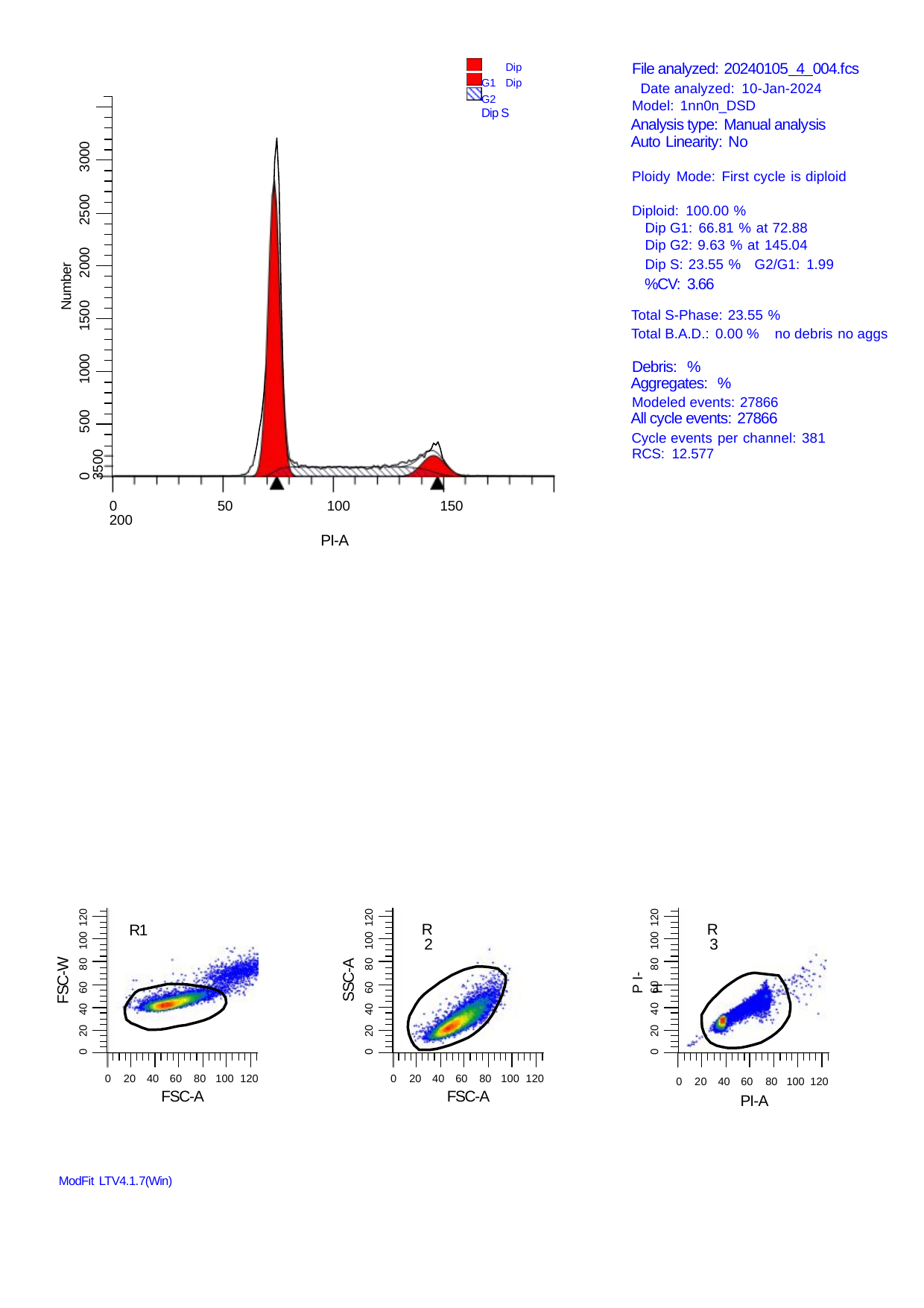

Dip G1 	Dip G2
File analyzed: 20240105 4 004.fcs Date analyzed: 10-Jan-2024
Model: 1nn0n_DSD
Analysis type: Manual analysis
Auto Linearity: No
Ploidy Mode: First cycle is diploid
Diploid: 100.00 %
Dip G1: 66.81 % at 72.88
Dip G2: 9.63 % at 145.04
Dip S: 23.55 % G2/G1: 1.99
%CV: 3.66
Total S-Phase: 23.55 %
Total B.A.D.: 0.00 % no debris no aggs
Debris: %
Aggregates: %
Modeled events: 27866
All cycle events: 27866
Cycle events per channel: 381
RCS: 12.577
	Dip S
Number
0 500 1000 1500 2000 2500 3000 3500
0 50 100 150 200
PI-A
R2
R3
R1
P I-H
SSC-A
FSC-W
0 20 40 60 80 100 120
0 20 40 60 80 100 120
0 20 40 60 80 100 120
0 20 40 60 80 100 120
FSC-A
0 20 40 60 80 100 120
FSC-A
0 20 40 60 80 100 120 PI-A
ModFit LTV4.1.7(Win)
