# Supplementary material for: Expression of GLOD4 in the Testis of the Qianbei Ma Goat and Its Effect on Leydig Cells
Source: Animals (Basel). 2024 Sep 8;14(17):2611. doi: 10.3390/ani14172611 (PMC11393997; doi:10.3390/ani14172611)
Supplement: Supplementary file 1 [file animals-14-02611-s001.zip › flow cytometry images/flow cytometry-pcNDNA3.1-NC.pptx]

## Slide 1
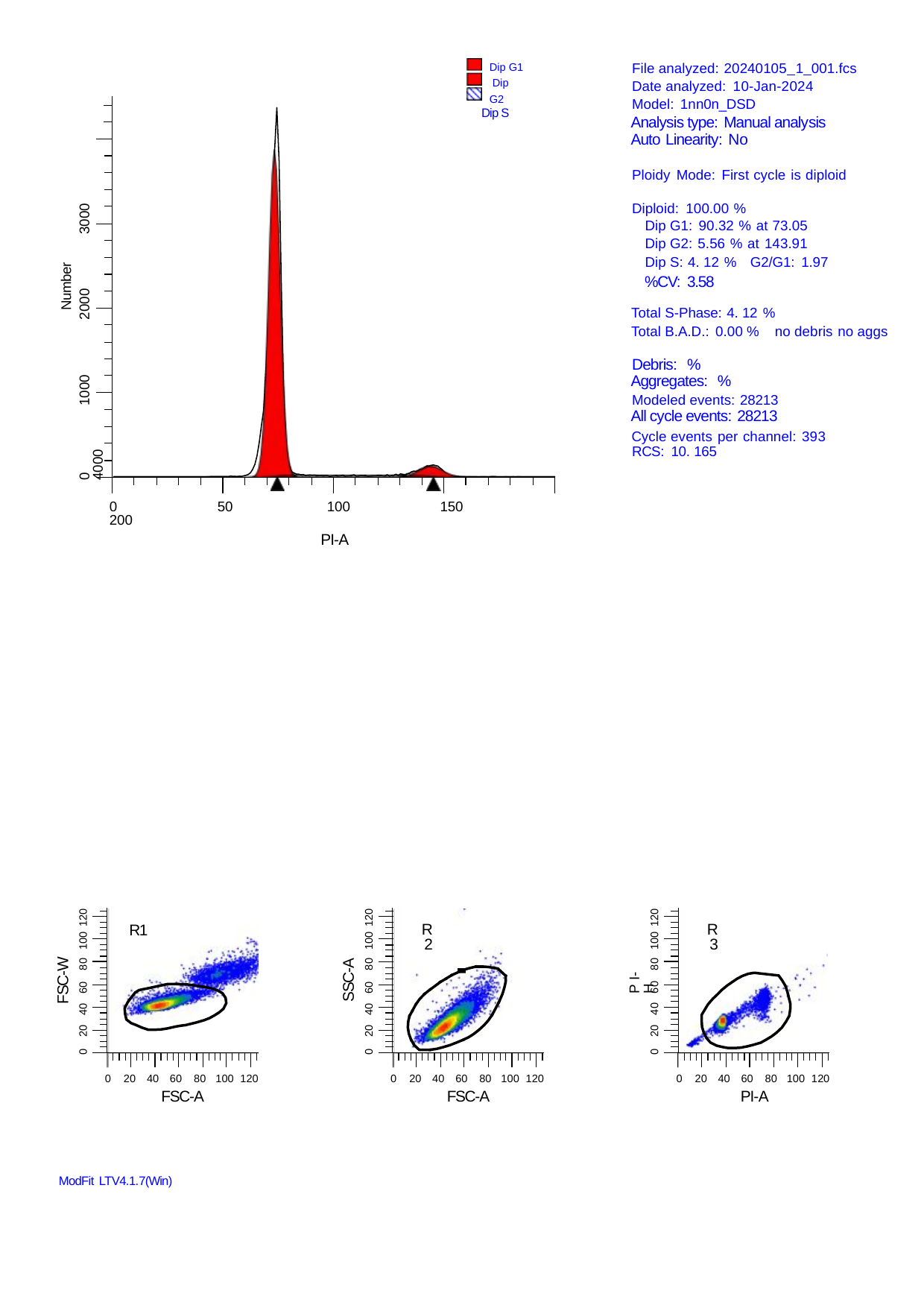

Dip G1 Dip G2
File analyzed: 20240105 1 001.fcs Date analyzed: 10-Jan-2024
Model: 1nn0n_DSD
Analysis type: Manual analysis
Auto Linearity: No
Ploidy Mode: First cycle is diploid
Diploid: 100.00 %
Dip G1: 90.32 % at 73.05
Dip G2: 5.56 % at 143.91
Dip S: 4. 12 % G2/G1: 1.97
%CV: 3.58
Total S-Phase: 4. 12 %
Total B.A.D.: 0.00 % no debris no aggs
Debris: %
Aggregates: %
Modeled events: 28213
All cycle events: 28213
Cycle events per channel: 393
RCS: 10. 165
	Dip S
Number
0 1000 2000 3000 4000
0 50 100 150 200
PI-A
R2
R3
R1
P I-H
SSC-A
FSC-W
0 20 40 60 80 100 120
0 20 40 60 80 100 120
0 20 40 60 80 100 120
0 20 40 60 80 100 120
FSC-A
0 20 40 60 80 100 120
FSC-A
0 20 40 60 80 100 120
PI-A
ModFit LTV4.1.7(Win)

## Slide 2
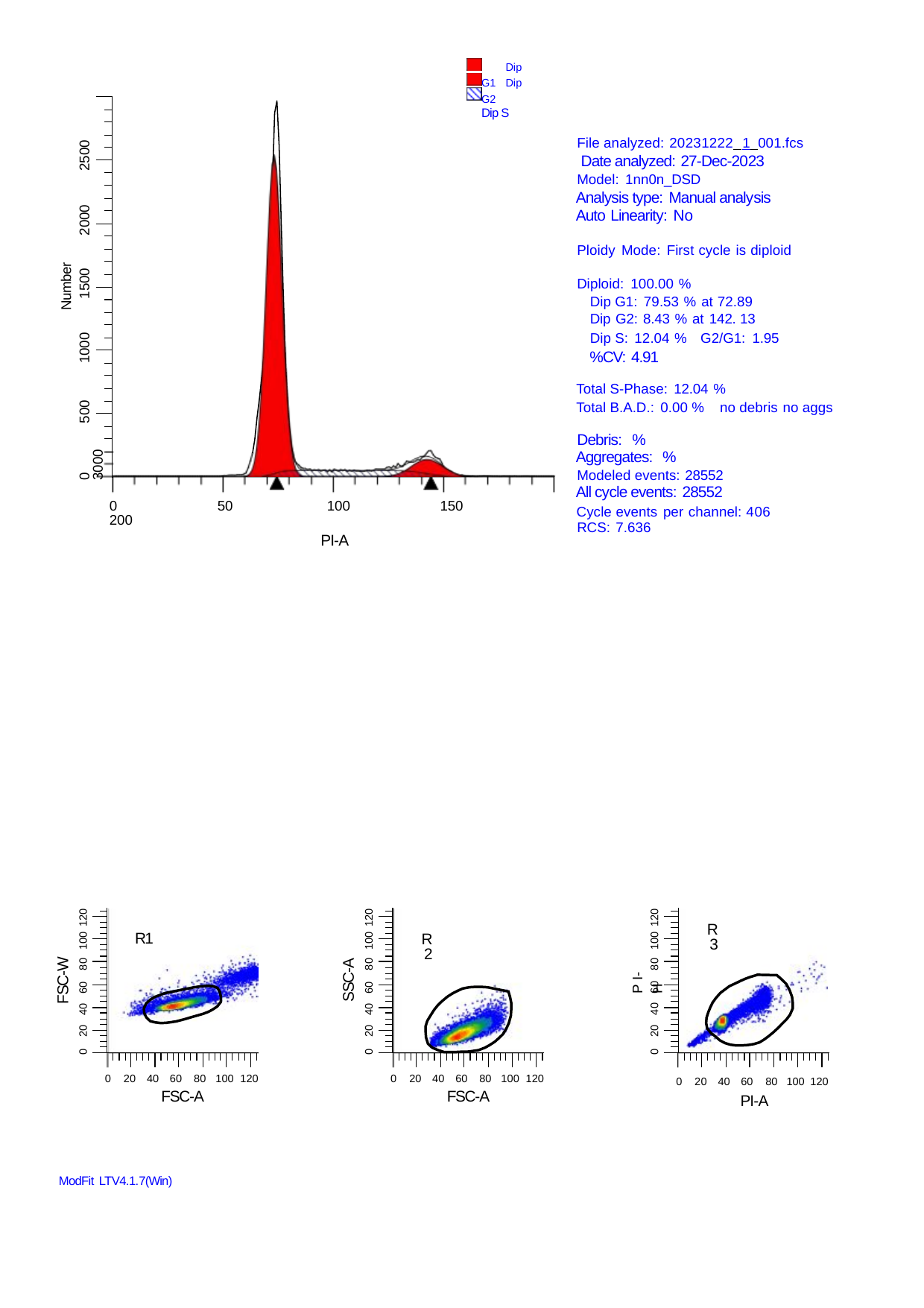

Dip G1 	Dip G2
	Dip S
File analyzed: 20231222 1 001.fcs Date analyzed: 27-Dec-2023
Model: 1nn0n_DSD
Analysis type: Manual analysis
Auto Linearity: No
Ploidy Mode: First cycle is diploid
Diploid: 100.00 %
Dip G1: 79.53 % at 72.89
Dip G2: 8.43 % at 142. 13
Dip S: 12.04 % G2/G1: 1.95
%CV: 4.91
Total S-Phase: 12.04 %
Total B.A.D.: 0.00 % no debris no aggs
Debris: %
Aggregates: %
Modeled events: 28552
All cycle events: 28552
Cycle events per channel: 406
RCS: 7.636
0 500 1000 1500 2000 2500 3000
Number
0 50 100 150 200
PI-A
R3
R1
R2
P I-H
SSC-A
FSC-W
0 20 40 60 80 100 120
0 20 40 60 80 100 120
0 20 40 60 80 100 120
0 20 40 60 80 100 120
FSC-A
0 20 40 60 80 100 120
FSC-A
0 20 40 60 80 100 120 PI-A
ModFit LTV4.1.7(Win)

## Slide 3
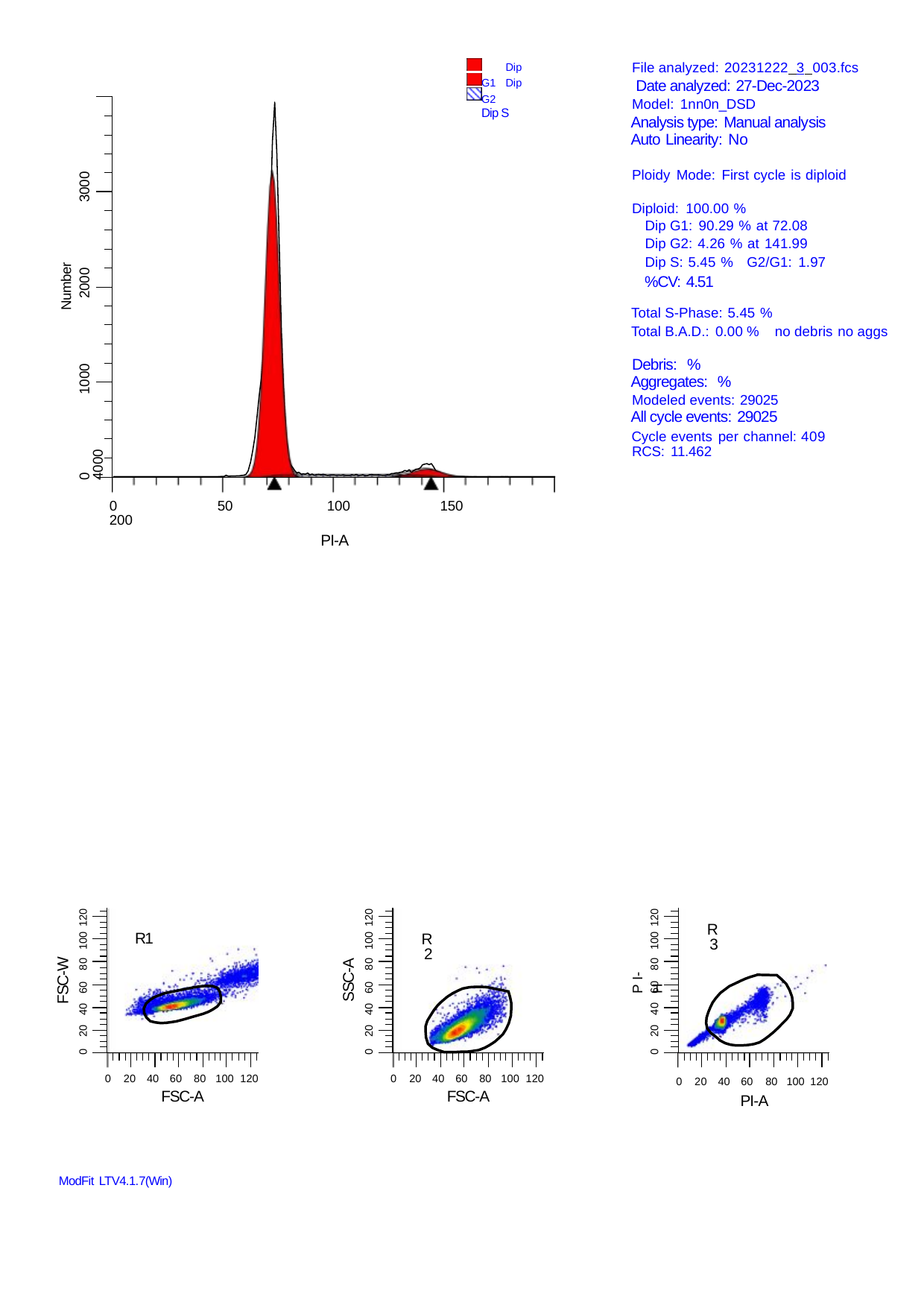

Dip G1 	Dip G2
File analyzed: 20231222 3 003.fcs Date analyzed: 27-Dec-2023
Model: 1nn0n_DSD
Analysis type: Manual analysis
Auto Linearity: No
Ploidy Mode: First cycle is diploid
Diploid: 100.00 %
Dip G1: 90.29 % at 72.08
Dip G2: 4.26 % at 141.99
Dip S: 5.45 % G2/G1: 1.97
%CV: 4.51
Total S-Phase: 5.45 %
Total B.A.D.: 0.00 % no debris no aggs
Debris: %
Aggregates: %
Modeled events: 29025
All cycle events: 29025
Cycle events per channel: 409
RCS: 11.462
	Dip S
0 1000 2000 3000 4000
Number
0 50 100 150 200
PI-A
R3
R1
R2
P I-H
SSC-A
FSC-W
0 20 40 60 80 100 120
0 20 40 60 80 100 120
0 20 40 60 80 100 120
0 20 40 60 80 100 120
FSC-A
0 20 40 60 80 100 120
FSC-A
0 20 40 60 80 100 120 PI-A
ModFit LTV4.1.7(Win)
