# Supplementary material for: Expression of GLOD4 in the Testis of the Qianbei Ma Goat and Its Effect on Leydig Cells
Source: Animals (Basel). 2024 Sep 8;14(17):2611. doi: 10.3390/ani14172611 (PMC11393997; doi:10.3390/ani14172611)
Supplement: Supplementary file 1 [file animals-14-02611-s001.zip › flow cytometry images/flow cytometry-sh-GLOD4.pptx]

## Slide 1
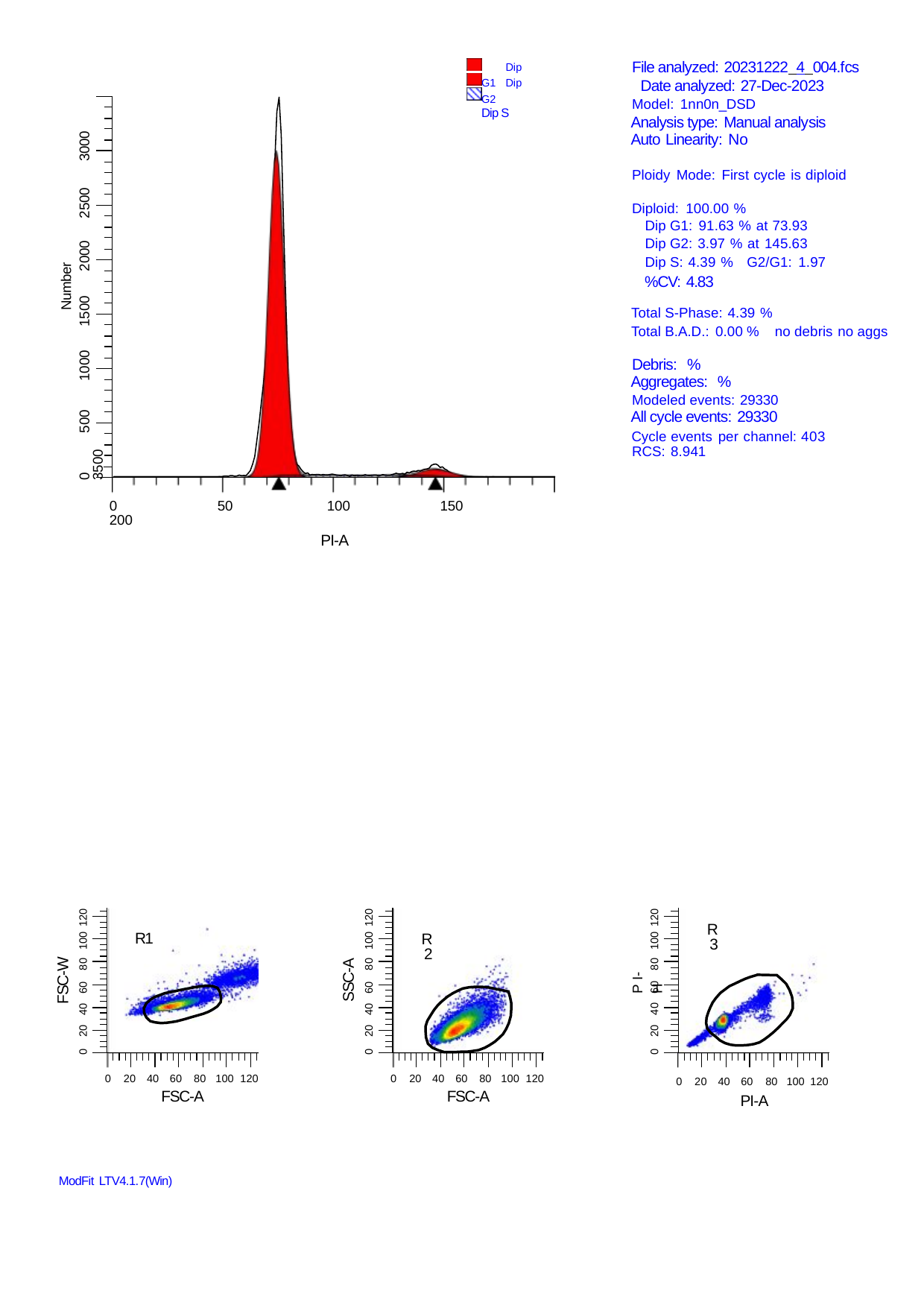

Dip G1 	Dip G2
File analyzed: 20231222 4 004.fcs Date analyzed: 27-Dec-2023
Model: 1nn0n_DSD
Analysis type: Manual analysis
Auto Linearity: No
Ploidy Mode: First cycle is diploid
Diploid: 100.00 %
Dip G1: 91.63 % at 73.93
Dip G2: 3.97 % at 145.63
Dip S: 4.39 % G2/G1: 1.97
%CV: 4.83
Total S-Phase: 4.39 %
Total B.A.D.: 0.00 % no debris no aggs
Debris: %
Aggregates: %
Modeled events: 29330
All cycle events: 29330
Cycle events per channel: 403
RCS: 8.941
	Dip S
0 500 1000 1500 2000 2500 3000 3500
Number
0 50 100 150 200
PI-A
R3
R1
R2
P I-H
SSC-A
FSC-W
0 20 40 60 80 100 120
0 20 40 60 80 100 120
0 20 40 60 80 100 120
0 20 40 60 80 100 120
FSC-A
0 20 40 60 80 100 120
FSC-A
0 20 40 60 80 100 120 PI-A
ModFit LTV4.1.7(Win)

## Slide 2
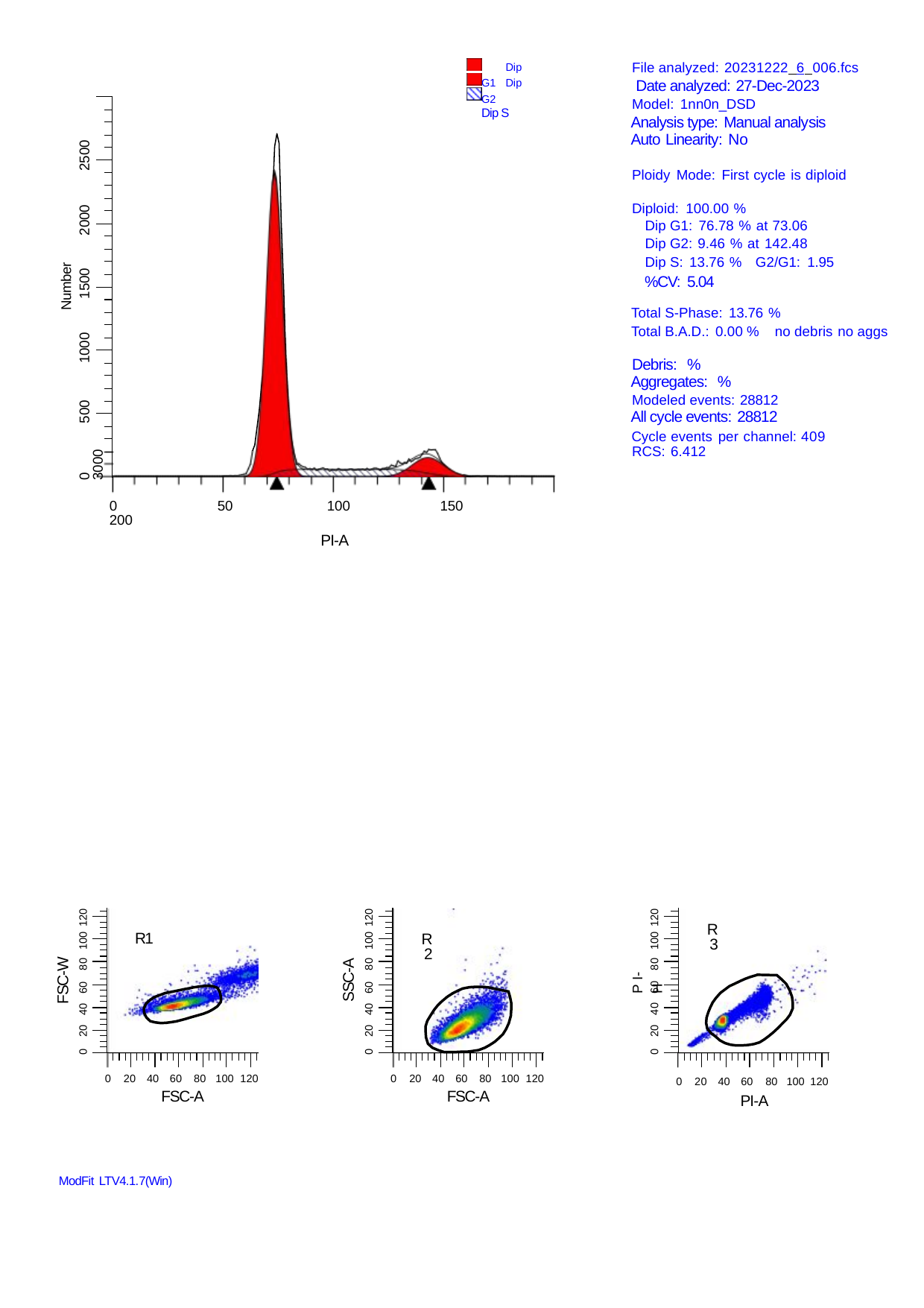

Dip G1 	Dip G2
File analyzed: 20231222 6 006.fcs Date analyzed: 27-Dec-2023
Model: 1nn0n_DSD
Analysis type: Manual analysis
Auto Linearity: No
Ploidy Mode: First cycle is diploid
Diploid: 100.00 %
Dip G1: 76.78 % at 73.06
Dip G2: 9.46 % at 142.48
Dip S: 13.76 % G2/G1: 1.95
%CV: 5.04
Total S-Phase: 13.76 %
Total B.A.D.: 0.00 % no debris no aggs
Debris: %
Aggregates: %
Modeled events: 28812
All cycle events: 28812
Cycle events per channel: 409
RCS: 6.412
	Dip S
0 500 1000 1500 2000 2500 3000
Number
0 50 100 150 200
PI-A
R3
R1
R2
P I-H
SSC-A
FSC-W
0 20 40 60 80 100 120
0 20 40 60 80 100 120
0 20 40 60 80 100 120
0 20 40 60 80 100 120
FSC-A
0 20 40 60 80 100 120
FSC-A
0 20 40 60 80 100 120 PI-A
ModFit LTV4.1.7(Win)

## Slide 3
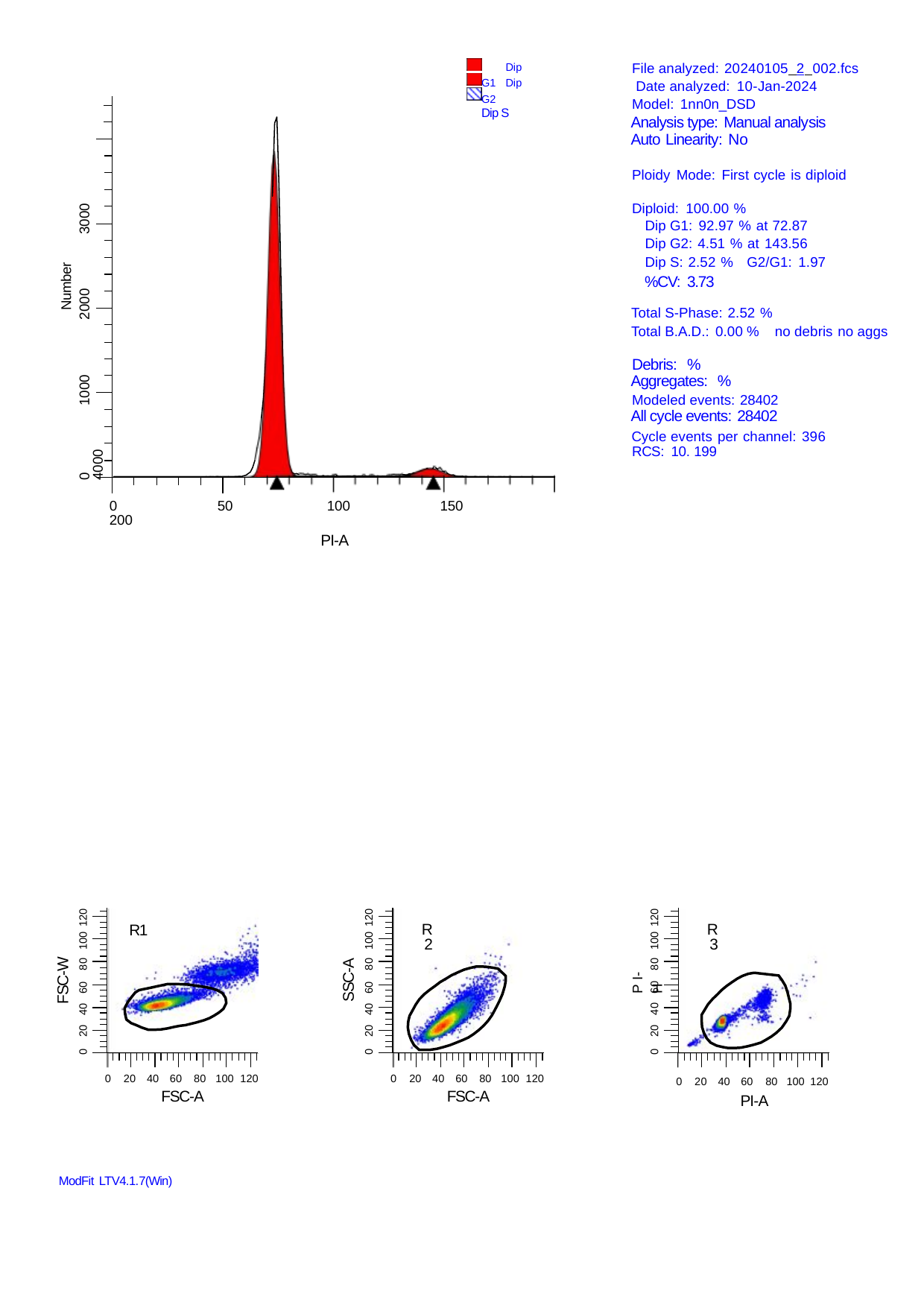

Dip G1 	Dip G2
File analyzed: 20240105 2 002.fcs Date analyzed: 10-Jan-2024
Model: 1nn0n_DSD
Analysis type: Manual analysis
Auto Linearity: No
Ploidy Mode: First cycle is diploid
Diploid: 100.00 %
Dip G1: 92.97 % at 72.87
Dip G2: 4.51 % at 143.56
Dip S: 2.52 % G2/G1: 1.97
%CV: 3.73
Total S-Phase: 2.52 %
Total B.A.D.: 0.00 % no debris no aggs
Debris: %
Aggregates: %
Modeled events: 28402
All cycle events: 28402
Cycle events per channel: 396
RCS: 10. 199
	Dip S
Number
0 1000 2000 3000 4000
0 50 100 150 200
PI-A
R2
R3
R1
P I-H
SSC-A
FSC-W
0 20 40 60 80 100 120
0 20 40 60 80 100 120
0 20 40 60 80 100 120
0 20 40 60 80 100 120
FSC-A
0 20 40 60 80 100 120
FSC-A
0 20 40 60 80 100 120 PI-A
ModFit LTV4.1.7(Win)
