# Supplementary material for: Expression of GLOD4 in the Testis of the Qianbei Ma Goat and Its Effect on Leydig Cells
Source: Animals (Basel). 2024 Sep 8;14(17):2611. doi: 10.3390/ani14172611 (PMC11393997; doi:10.3390/ani14172611)
Supplement: Supplementary file 1 [file animals-14-02611-s001.zip › flow cytometry images/flow cytometry-sh-NC.pptx]

## Slide 1
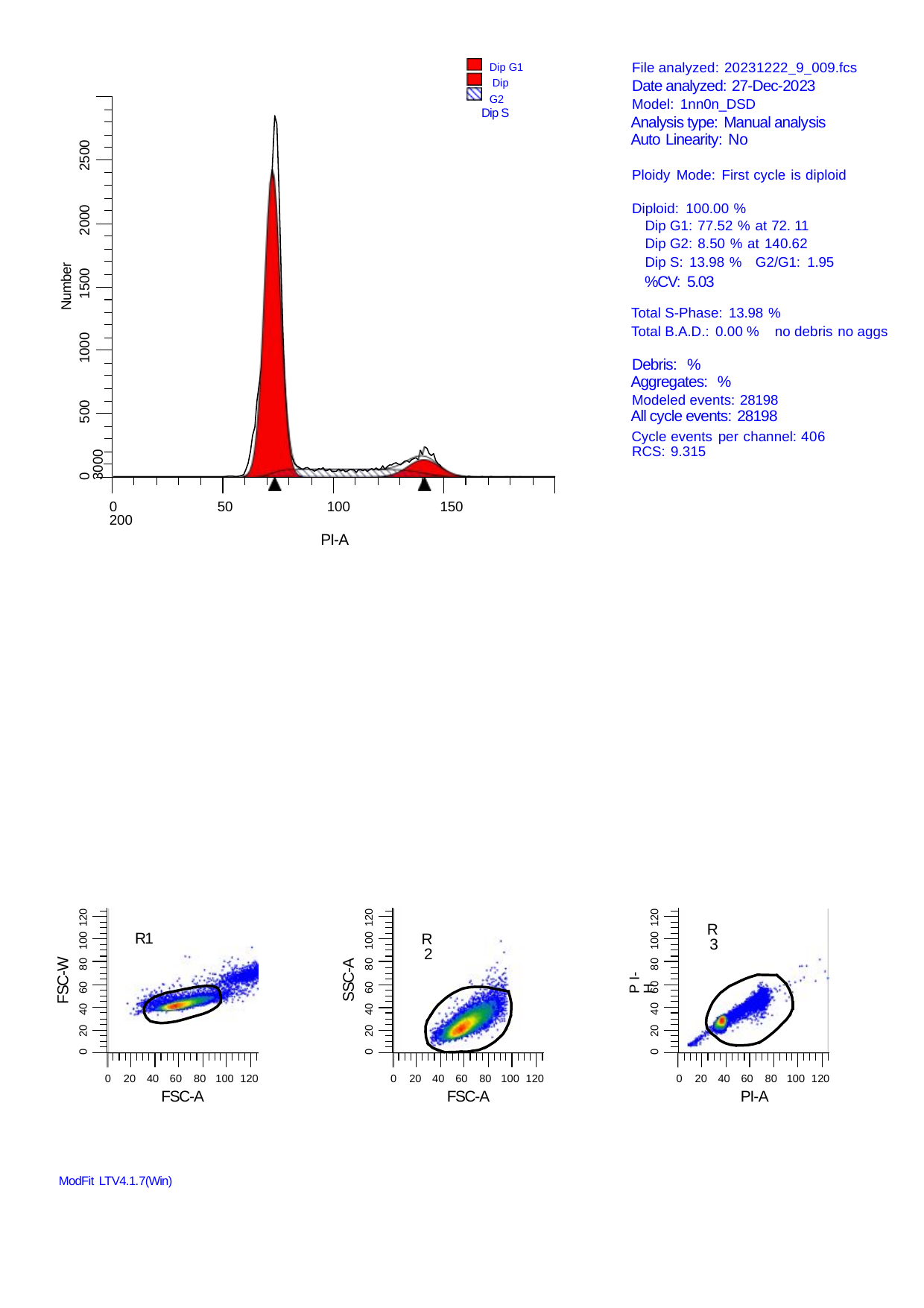

Dip G1 Dip G2
File analyzed: 20231222 9 009.fcs Date analyzed: 27-Dec-2023
Model: 1nn0n_DSD
Analysis type: Manual analysis
Auto Linearity: No
Ploidy Mode: First cycle is diploid
Diploid: 100.00 %
Dip G1: 77.52 % at 72. 11
Dip G2: 8.50 % at 140.62
Dip S: 13.98 % G2/G1: 1.95
%CV: 5.03
Total S-Phase: 13.98 %
Total B.A.D.: 0.00 % no debris no aggs
Debris: %
Aggregates: %
Modeled events: 28198
All cycle events: 28198
Cycle events per channel: 406
RCS: 9.315
	Dip S
0 500 1000 1500 2000 2500 3000
Number
0 50 100 150 200
PI-A
R3
R1
R2
P I-H
SSC-A
FSC-W
0 20 40 60 80 100 120
0 20 40 60 80 100 120
0 20 40 60 80 100 120
0 20 40 60 80 100 120
FSC-A
0 20 40 60 80 100 120
FSC-A
0 20 40 60 80 100 120
PI-A
ModFit LTV4.1.7(Win)

## Slide 2
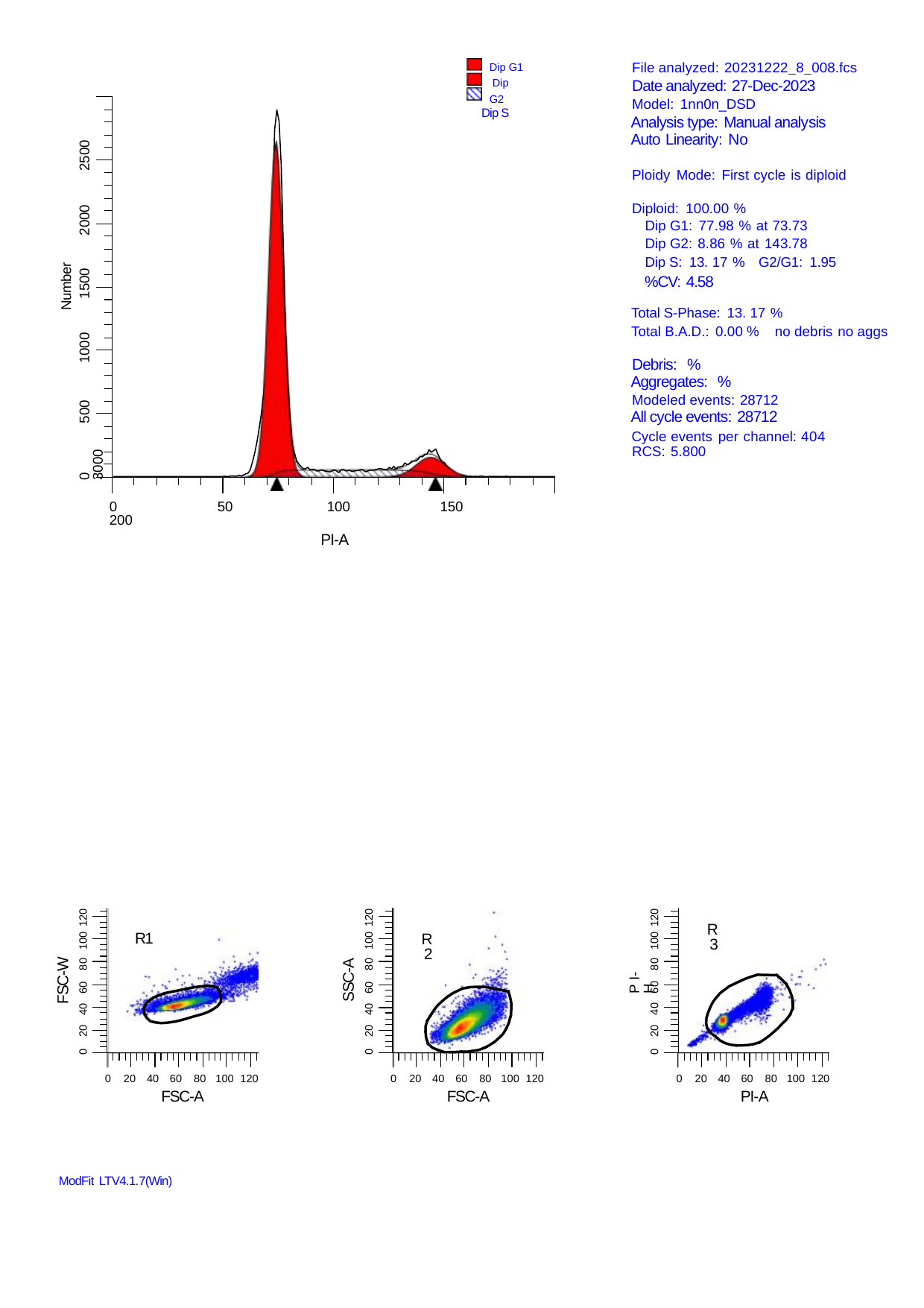

Dip G1 Dip G2
File analyzed: 20231222 8 008.fcs Date analyzed: 27-Dec-2023
Model: 1nn0n_DSD
Analysis type: Manual analysis
Auto Linearity: No
Ploidy Mode: First cycle is diploid
Diploid: 100.00 %
Dip G1: 77.98 % at 73.73
Dip G2: 8.86 % at 143.78
Dip S: 13. 17 % G2/G1: 1.95
%CV: 4.58
Total S-Phase: 13. 17 %
Total B.A.D.: 0.00 % no debris no aggs
Debris: %
Aggregates: %
Modeled events: 28712
All cycle events: 28712
Cycle events per channel: 404
RCS: 5.800
	Dip S
0 500 1000 1500 2000 2500 3000
Number
0 50 100 150 200
PI-A
R3
R1
R2
P I-H
SSC-A
FSC-W
0 20 40 60 80 100 120
0 20 40 60 80 100 120
0 20 40 60 80 100 120
0 20 40 60 80 100 120
FSC-A
0 20 40 60 80 100 120
FSC-A
0 20 40 60 80 100 120
PI-A
ModFit LTV4.1.7(Win)

## Slide 3
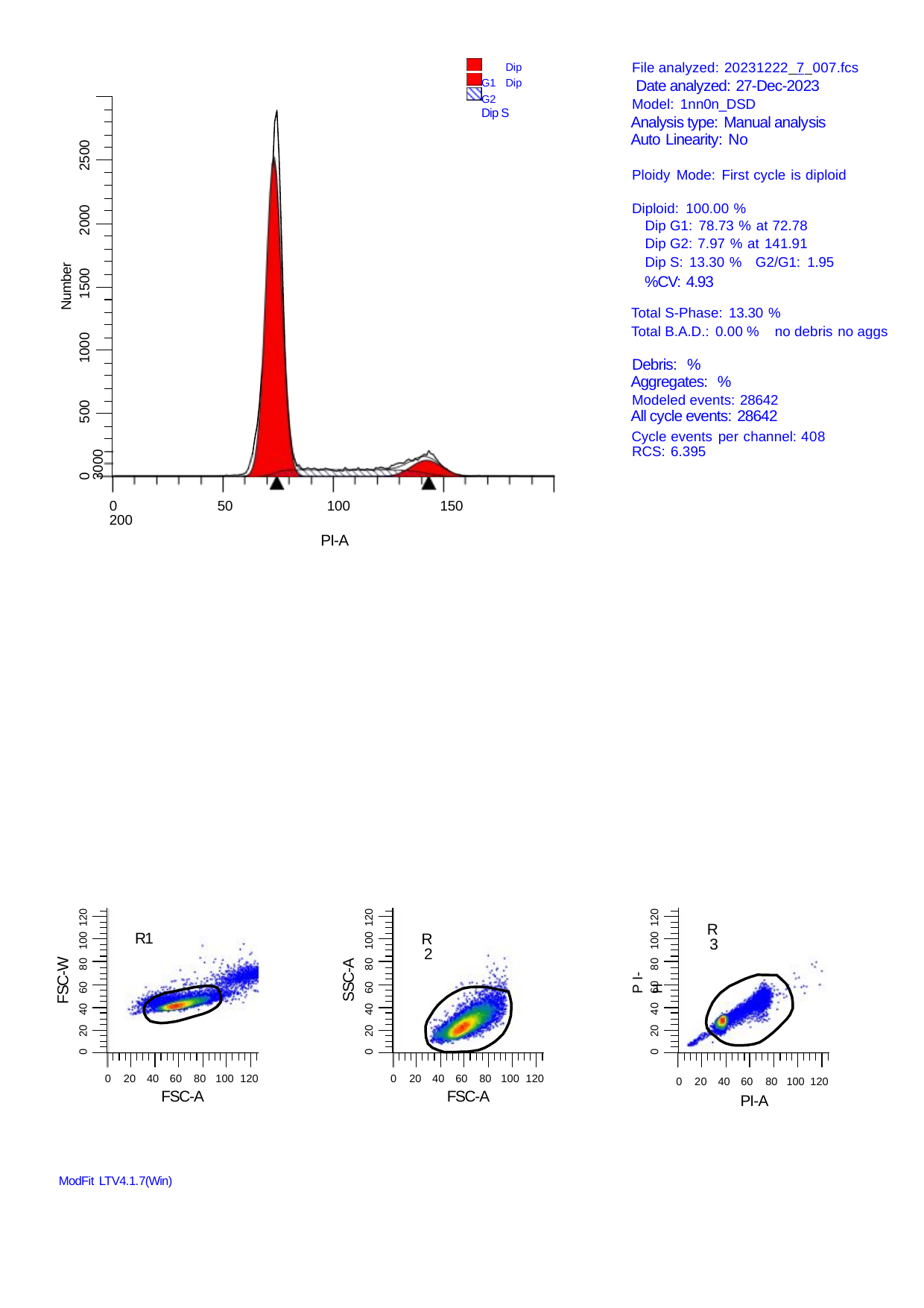

Dip G1 	Dip G2
File analyzed: 20231222 7 007.fcs Date analyzed: 27-Dec-2023
Model: 1nn0n_DSD
Analysis type: Manual analysis
Auto Linearity: No
Ploidy Mode: First cycle is diploid
Diploid: 100.00 %
Dip G1: 78.73 % at 72.78
Dip G2: 7.97 % at 141.91
Dip S: 13.30 % G2/G1: 1.95
%CV: 4.93
Total S-Phase: 13.30 %
Total B.A.D.: 0.00 % no debris no aggs
Debris: %
Aggregates: %
Modeled events: 28642
All cycle events: 28642
Cycle events per channel: 408
RCS: 6.395
	Dip S
0 500 1000 1500 2000 2500 3000
Number
0 50 100 150 200
PI-A
R3
R1
R2
P I-H
SSC-A
FSC-W
0 20 40 60 80 100 120
0 20 40 60 80 100 120
0 20 40 60 80 100 120
0 20 40 60 80 100 120
FSC-A
0 20 40 60 80 100 120
FSC-A
0 20 40 60 80 100 120 PI-A
ModFit LTV4.1.7(Win)
