# Supplementary material for: Expression of GLOD4 in the Testis of the Qianbei Ma Goat and Its Effect on Leydig Cells
Source: Animals (Basel). 2024 Sep 8;14(17):2611. doi: 10.3390/ani14172611 (PMC11393997; doi:10.3390/ani14172611)

## Slide 1
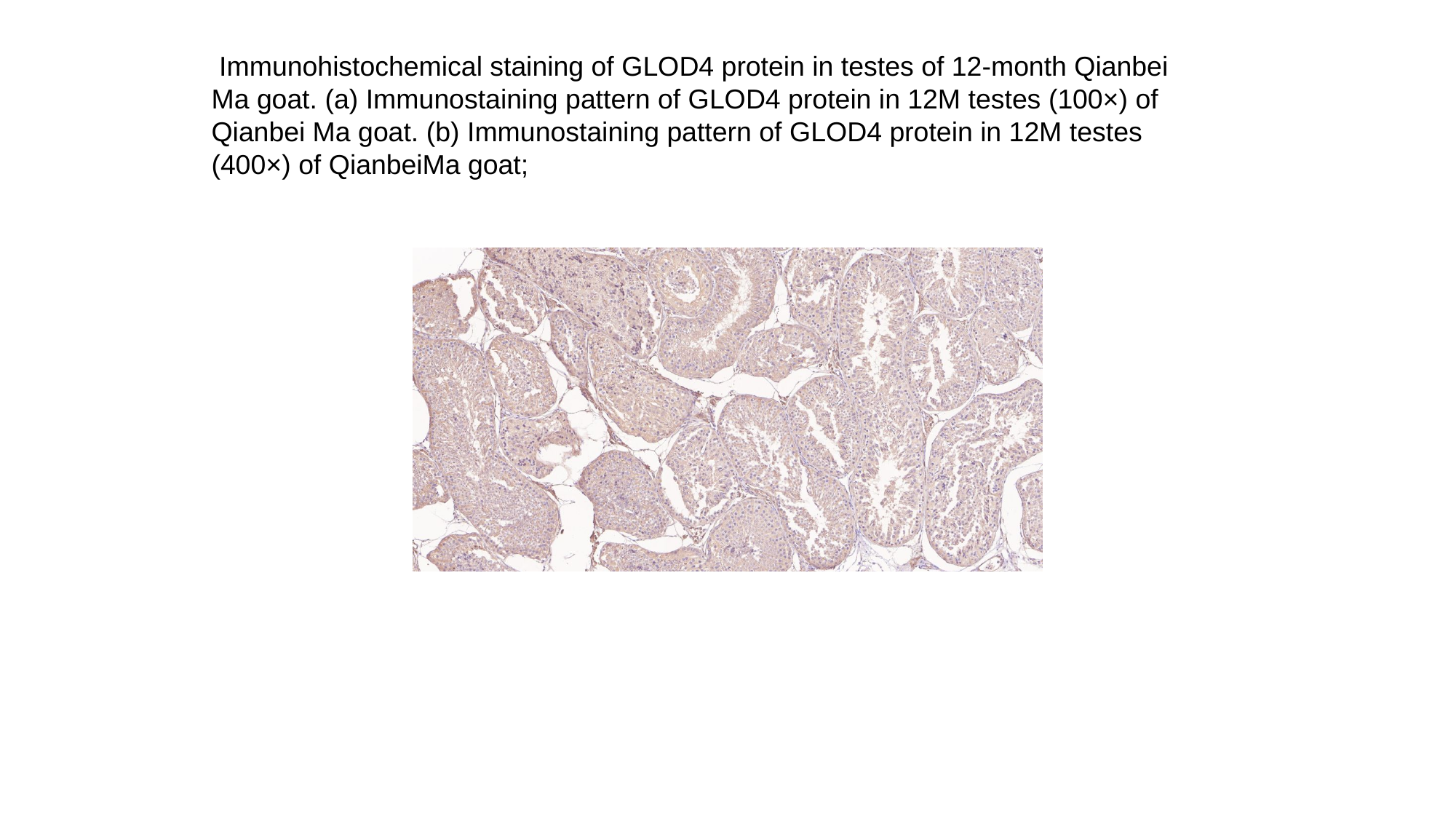

Immunohistochemical staining of GLOD4 protein in testes of 12-month Qianbei Ma goat. (a) Immunostaining pattern of GLOD4 protein in 12M testes (100×) of Qianbei Ma goat. (b) Immunostaining pattern of GLOD4 protein in 12M testes (400×) of QianbeiMa goat;

## Slide 2
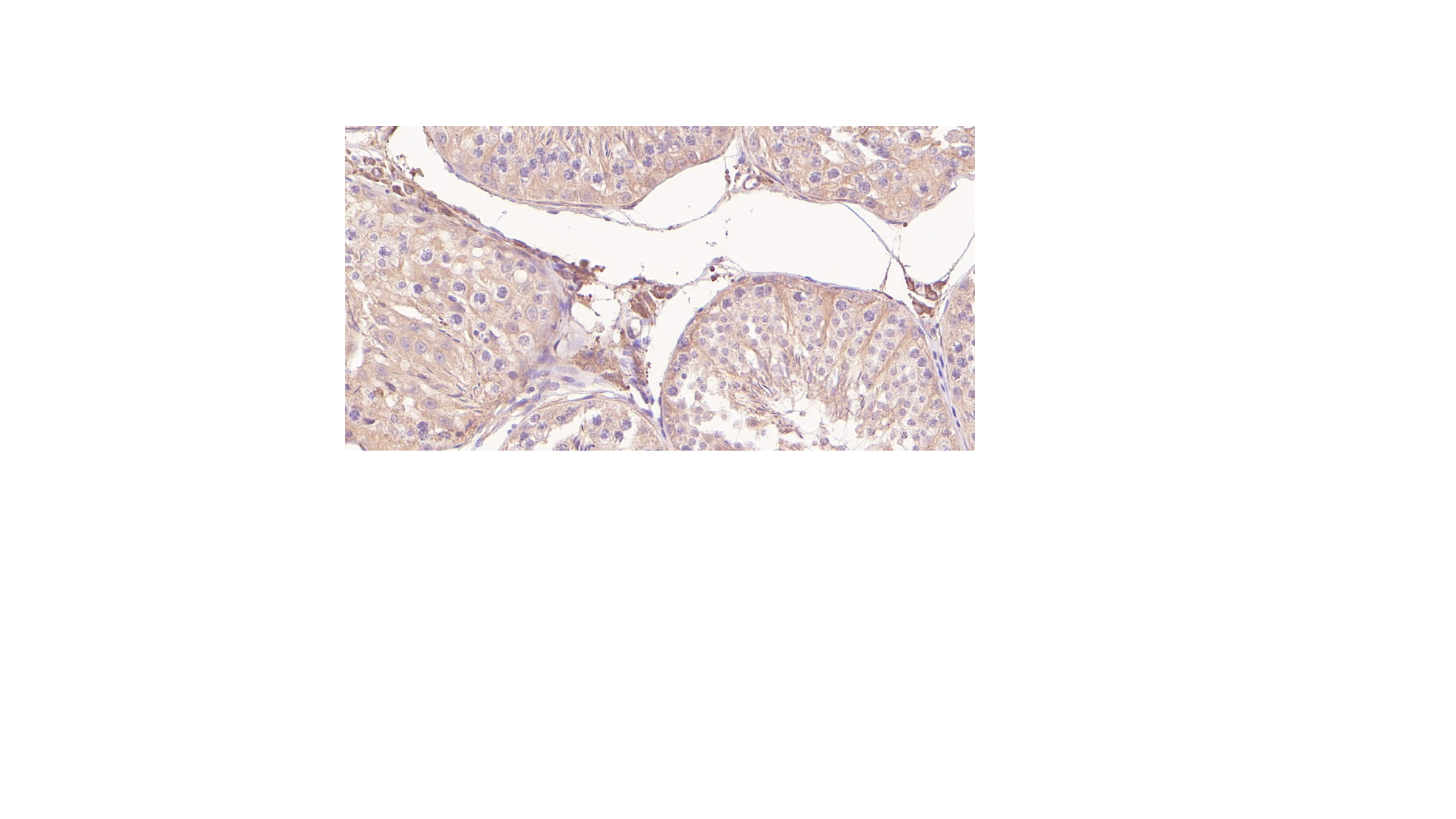

## Slide 3
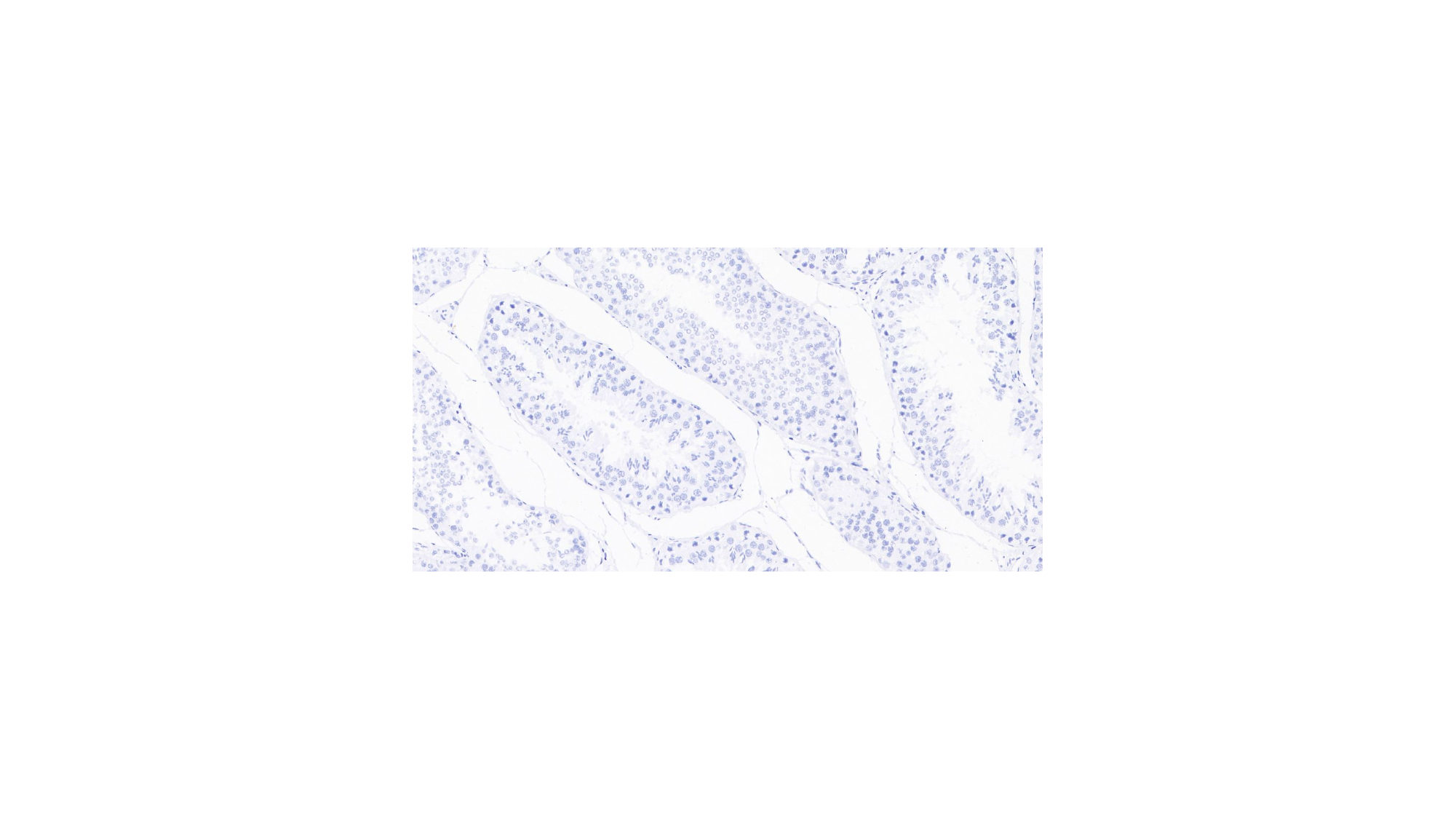

Supplement: Supplementary file 1 [file animals-14-02611-s001.zip › Immunohistochemistry images.pptx]
