# Supplementary figures and images for: Expression of GLOD4 in the Testis of the Qianbei Ma Goat and Its Effect on Leydig Cells
Source: Animals (Basel). 2024 Sep 8;14(17):2611. doi: 10.3390/ani14172611 (PMC11393997; doi:10.3390/ani14172611)

## Slide 1
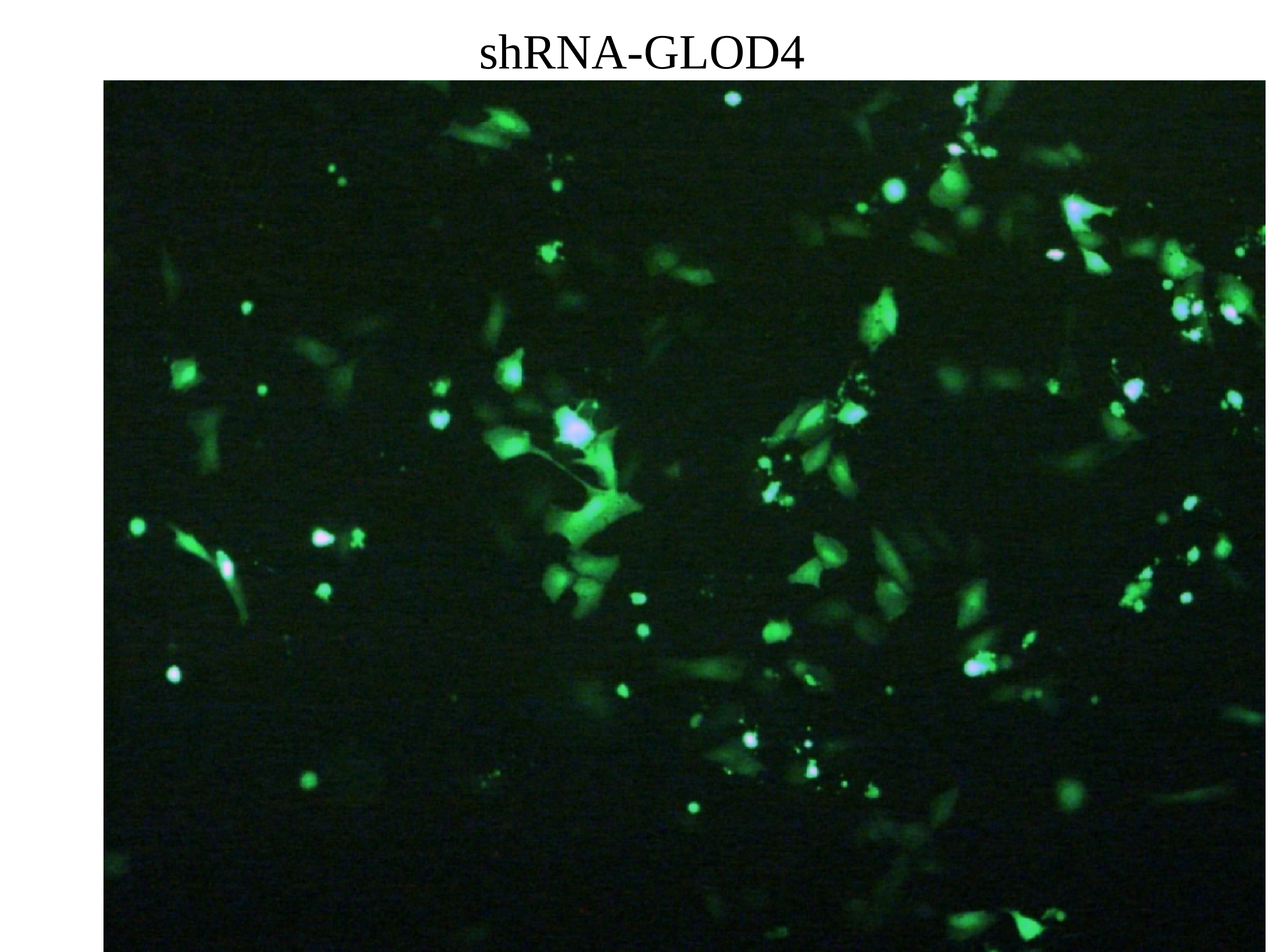

shRNA-GLOD4

## Slide 2
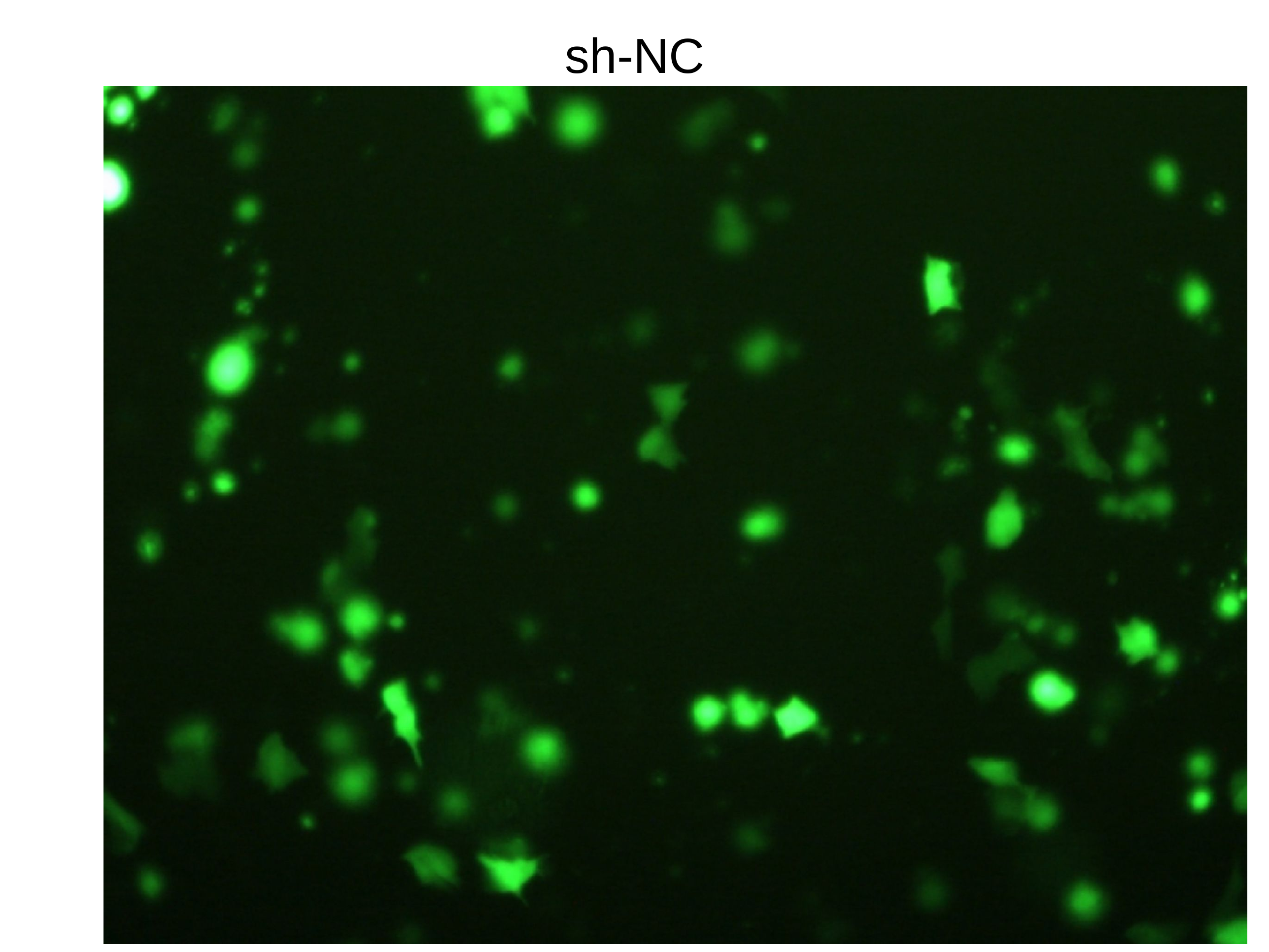

sh-NC

## Slide 3
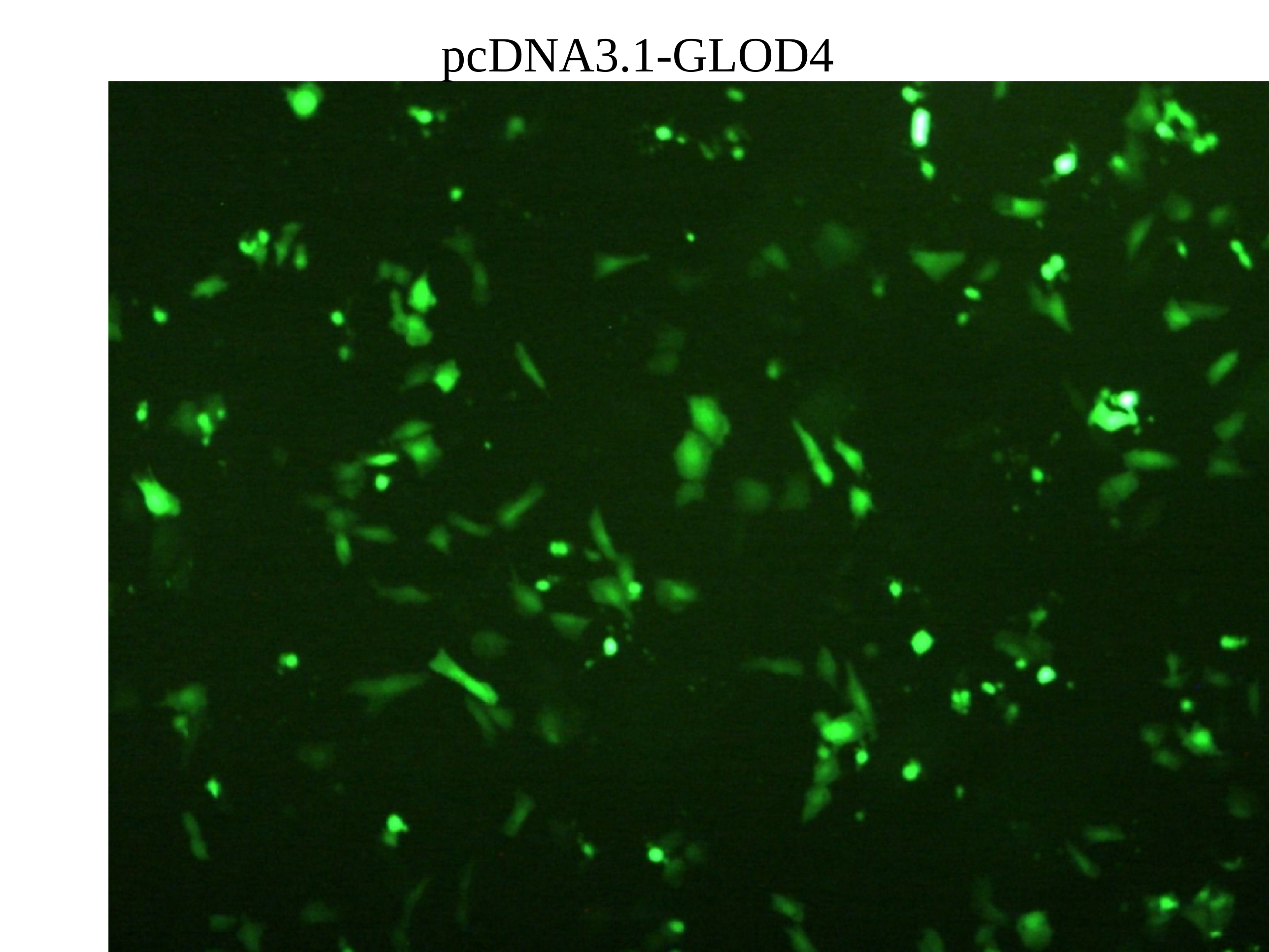

pcDNA3.1-GLOD4

## Slide 4
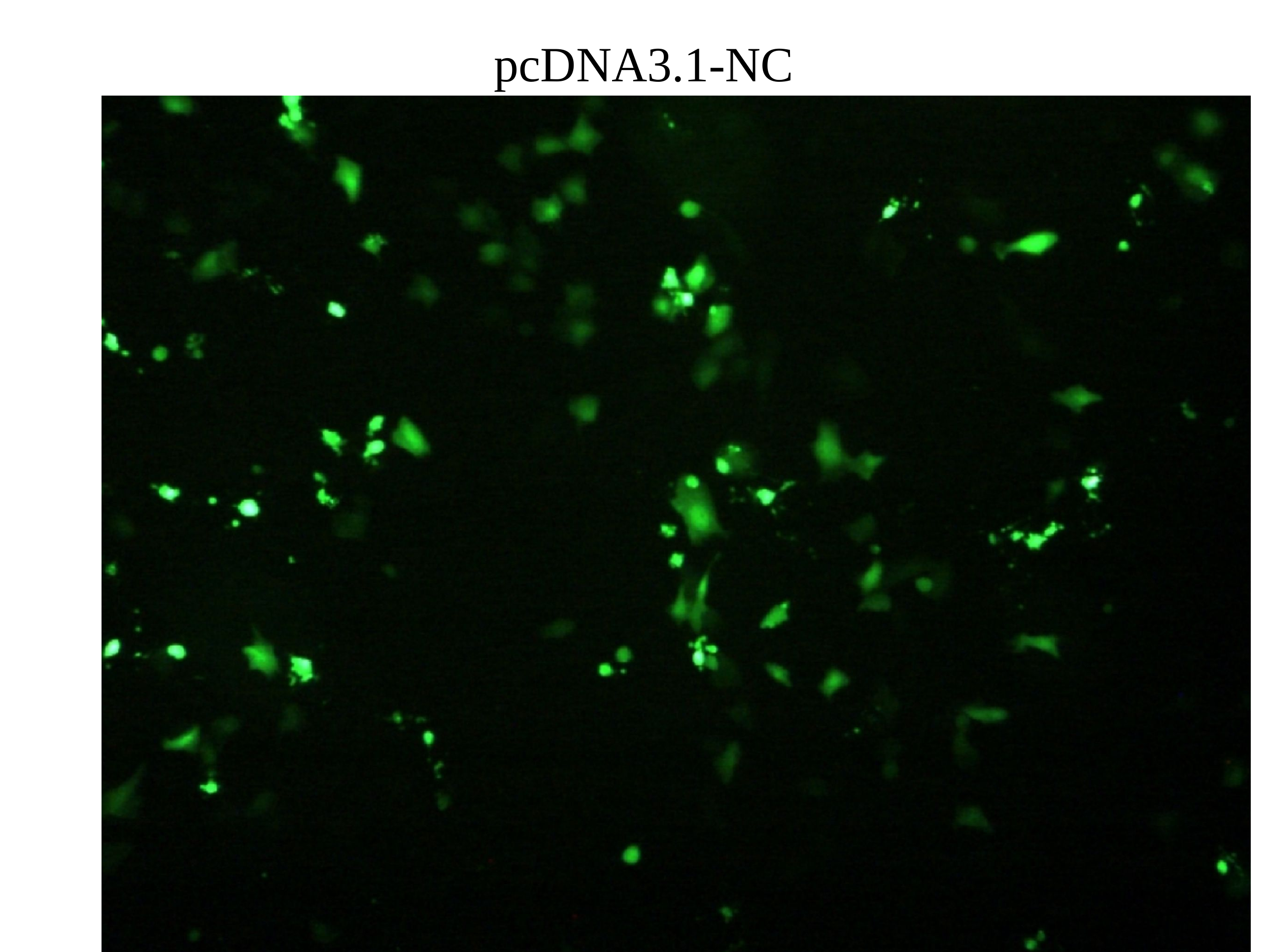

pcDNA3.1-NC

Supplement: Supplementary file 1 [file animals-14-02611-s001.zip › Plasmid transfection.pptx]

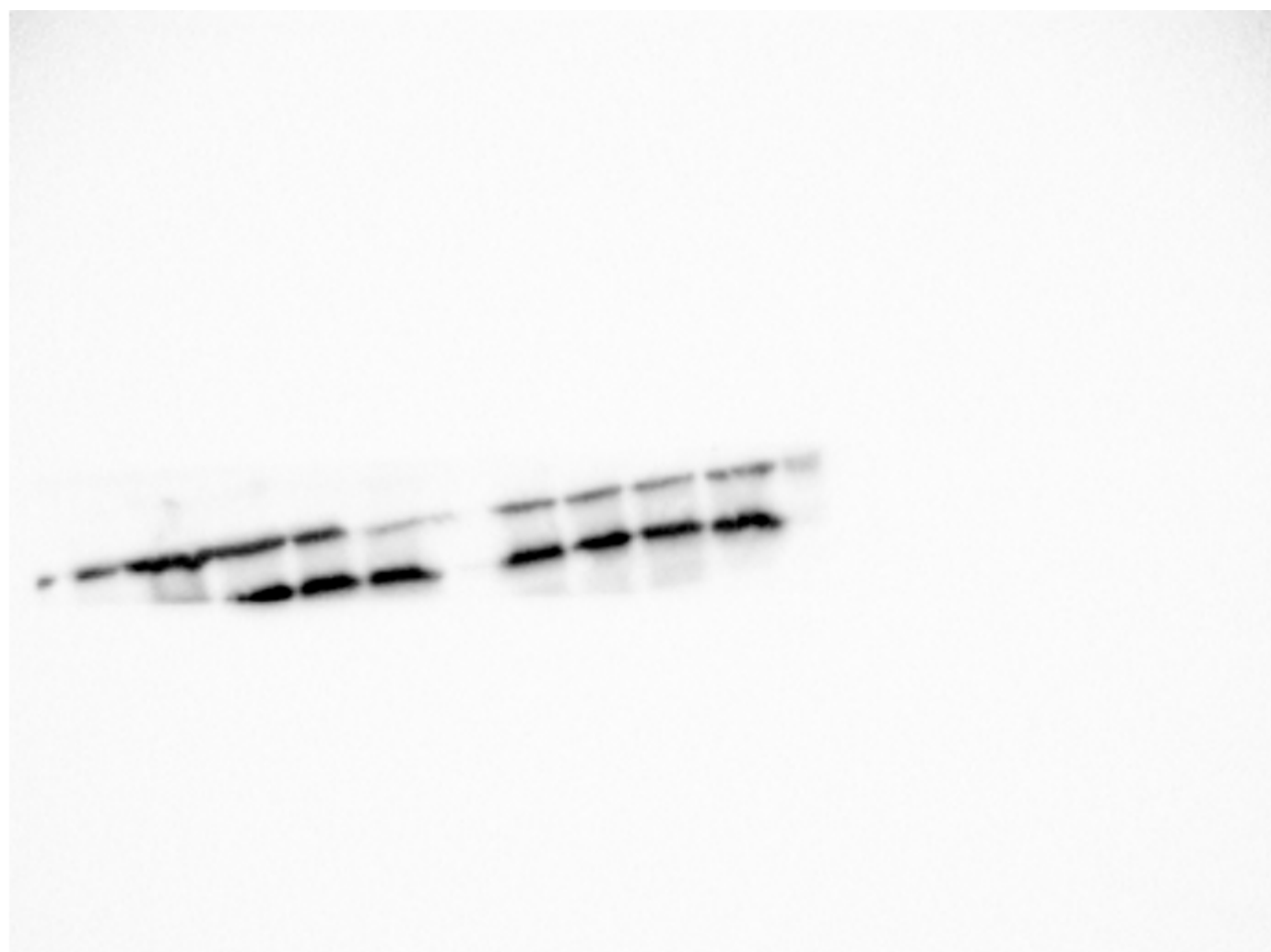

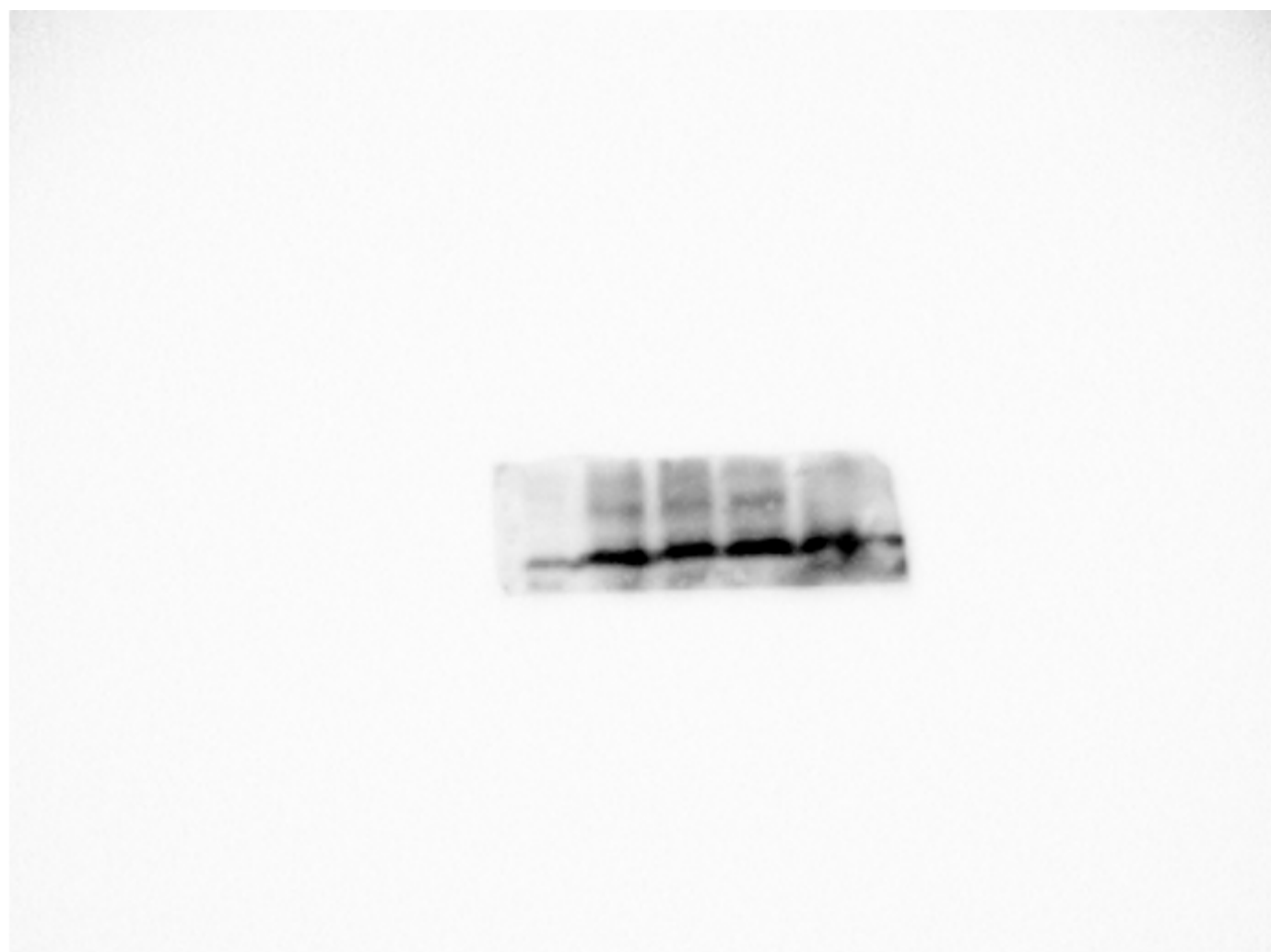

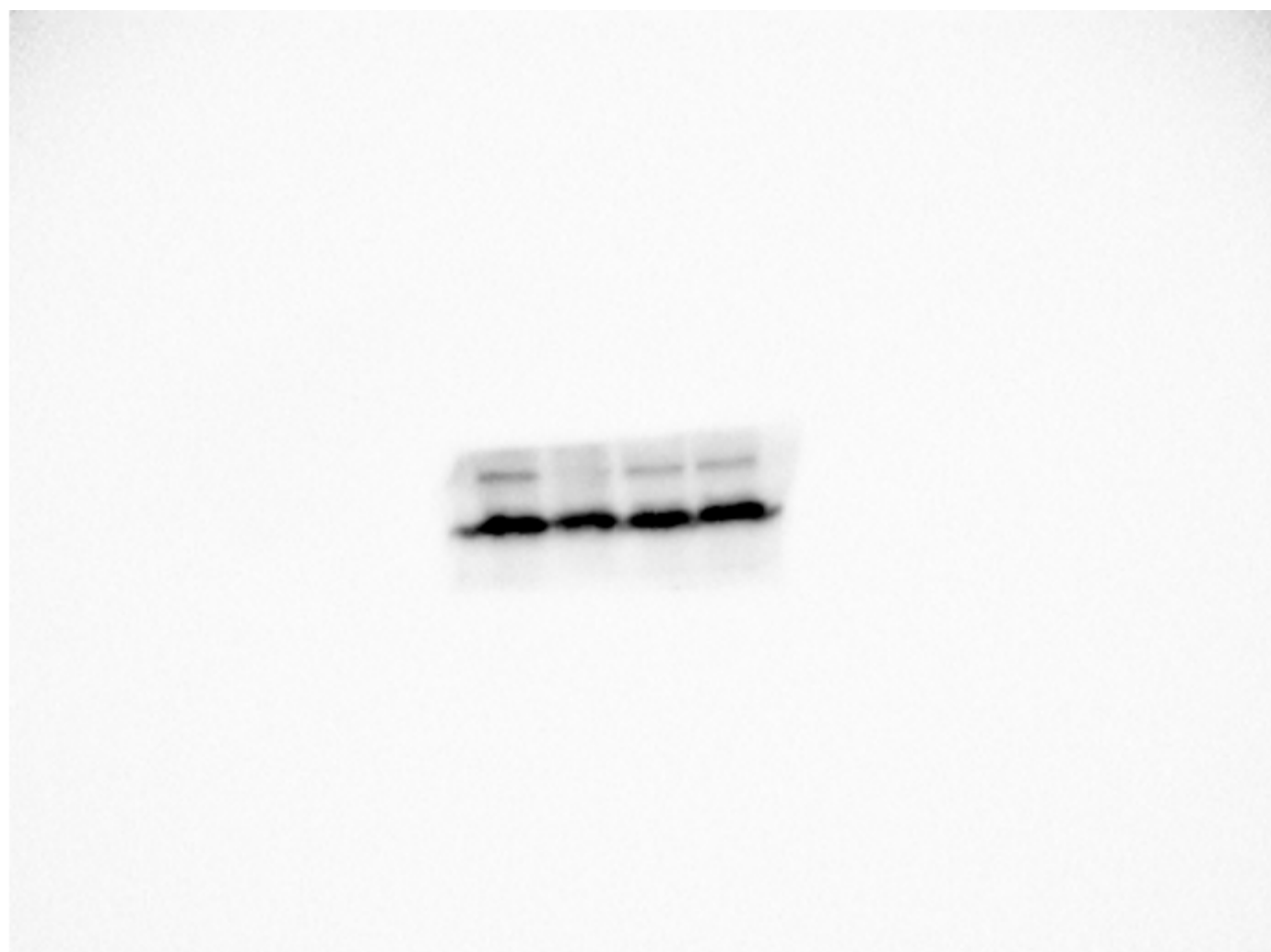

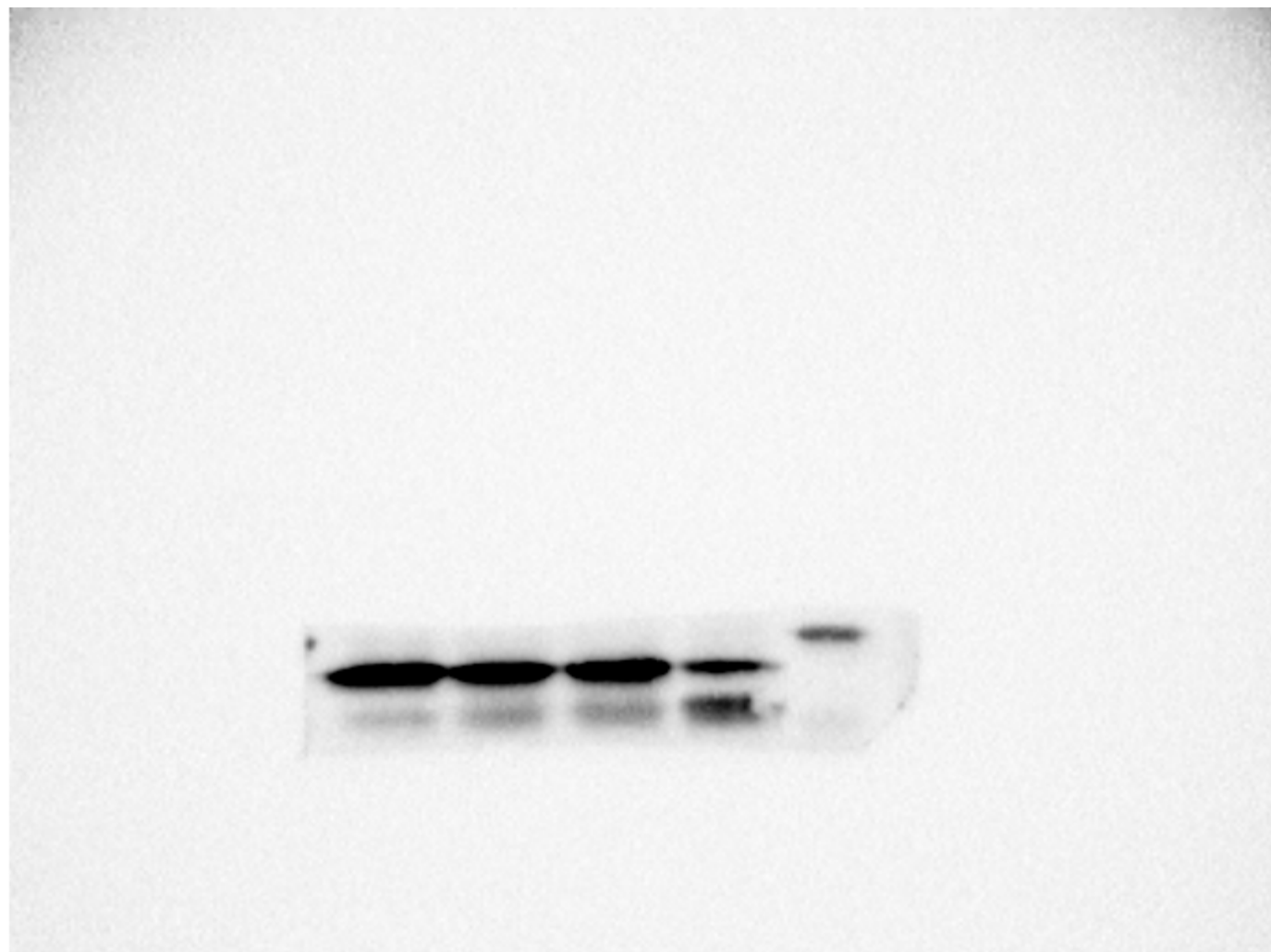

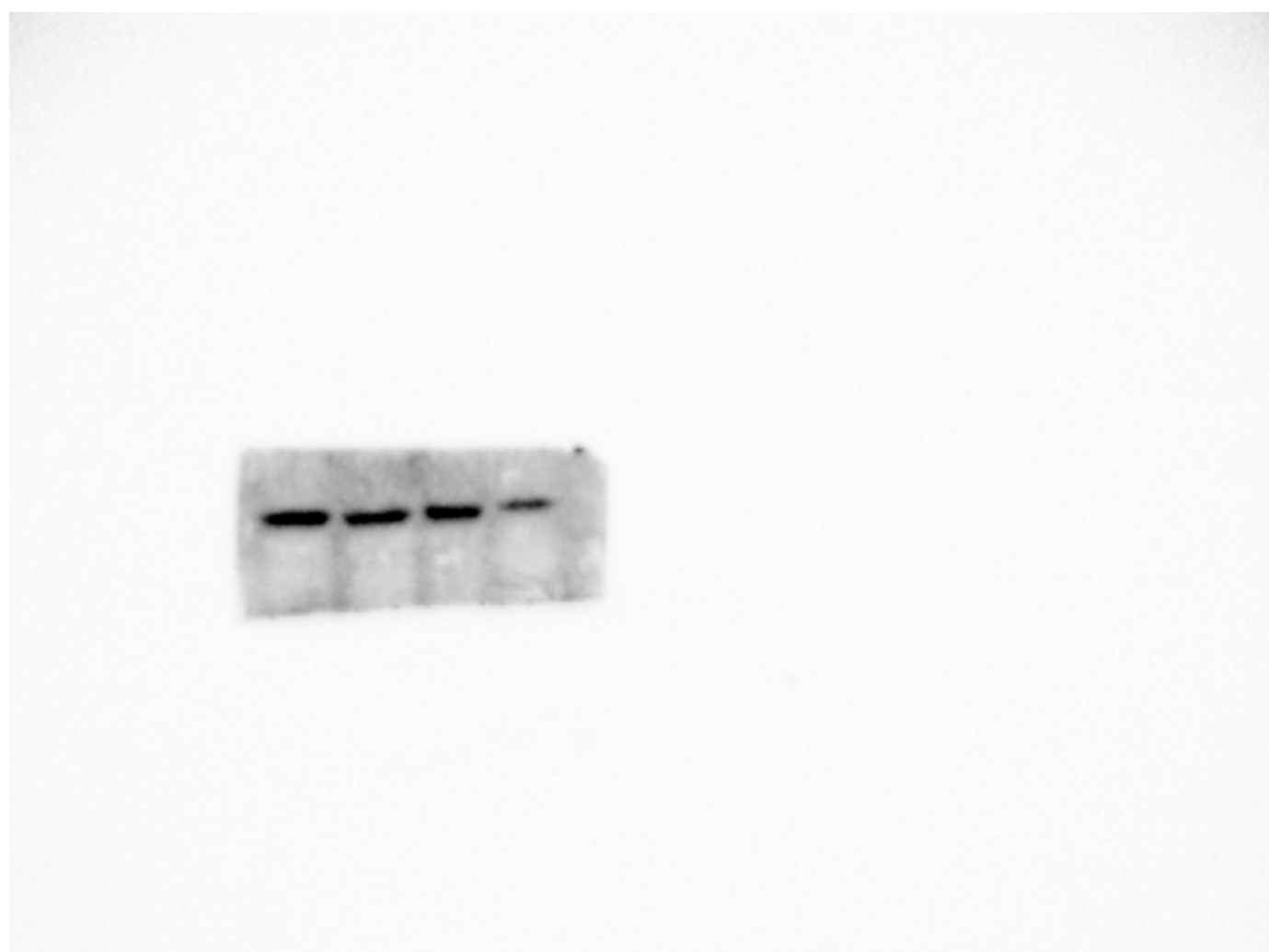

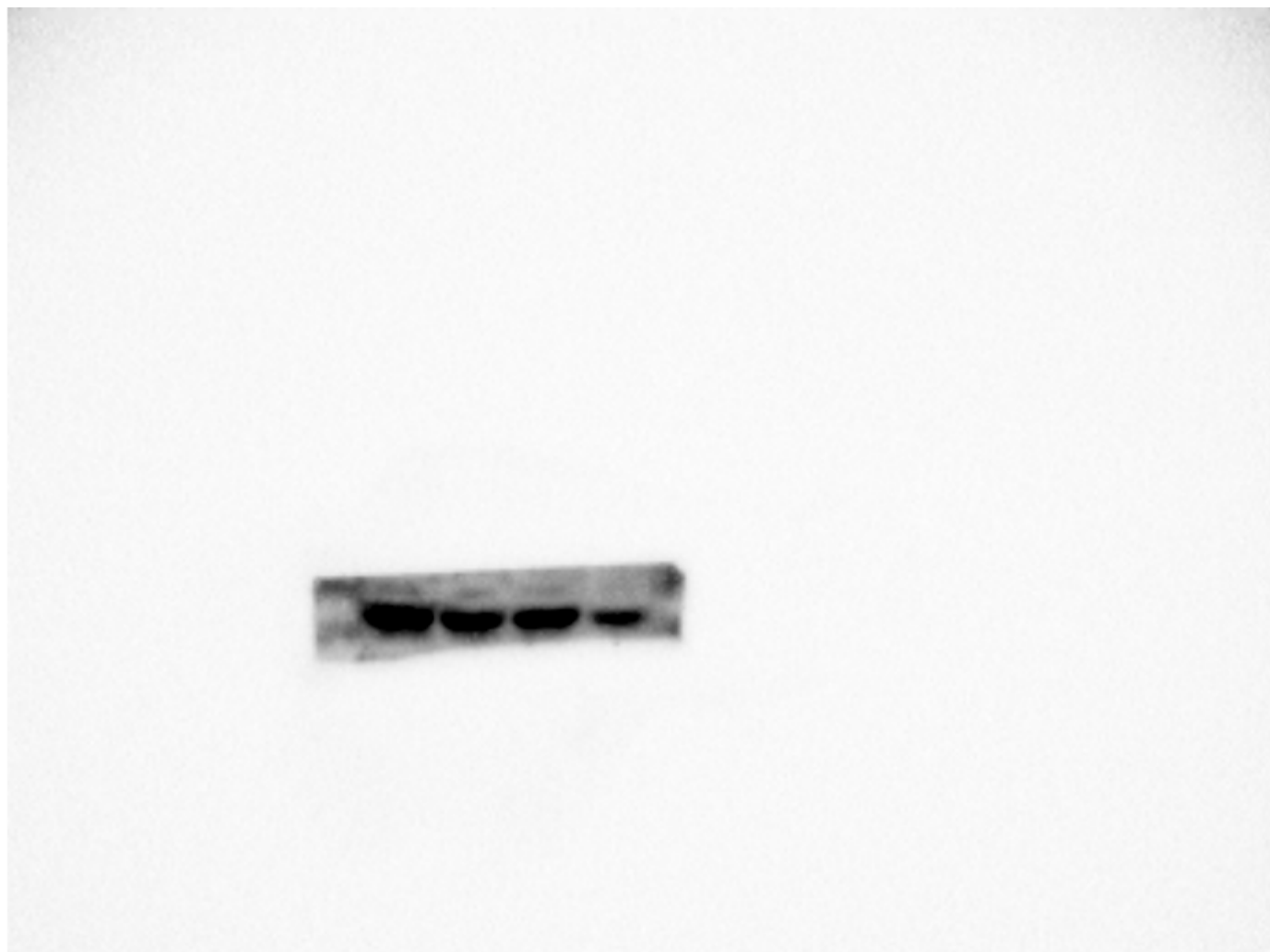

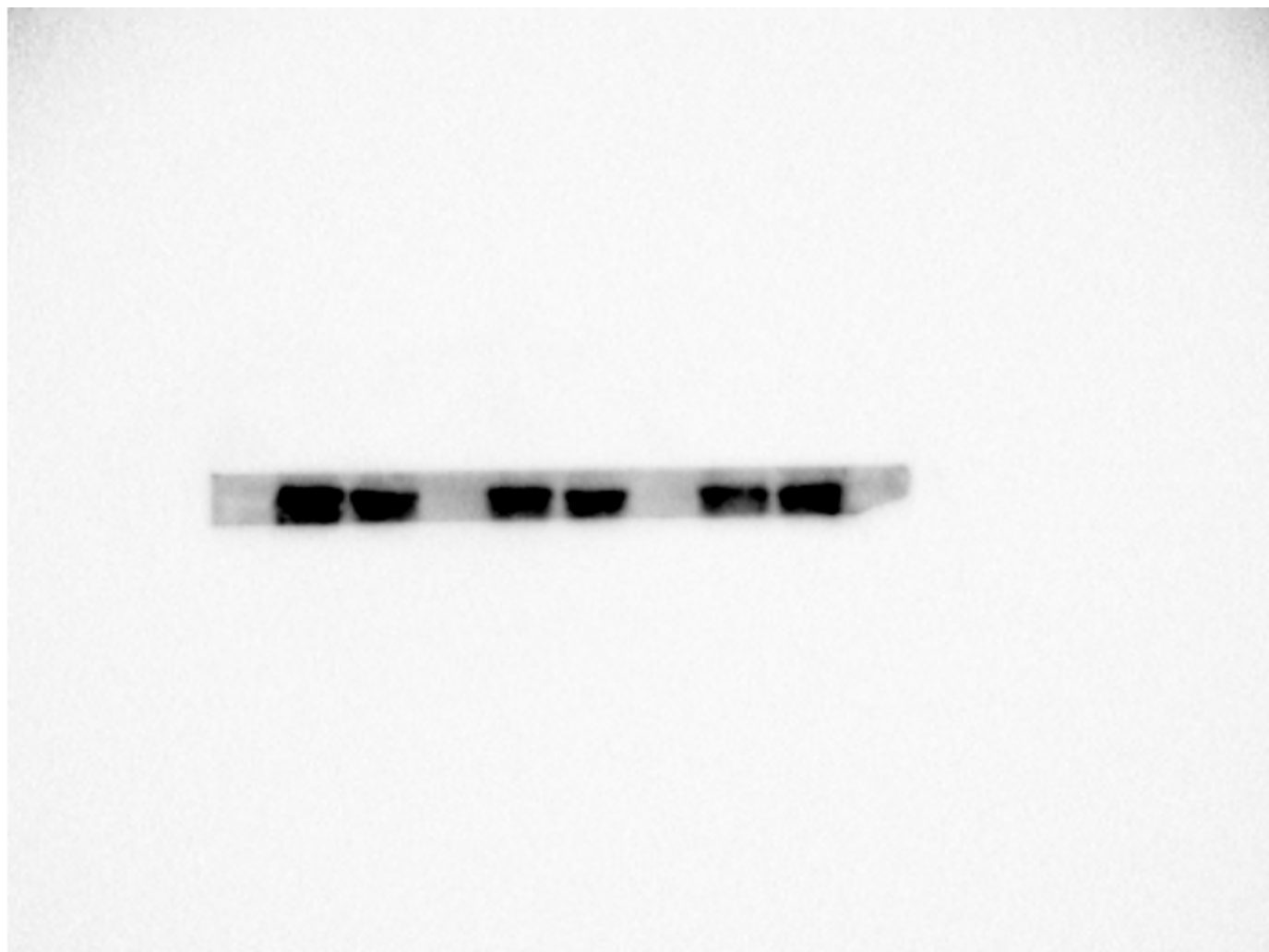

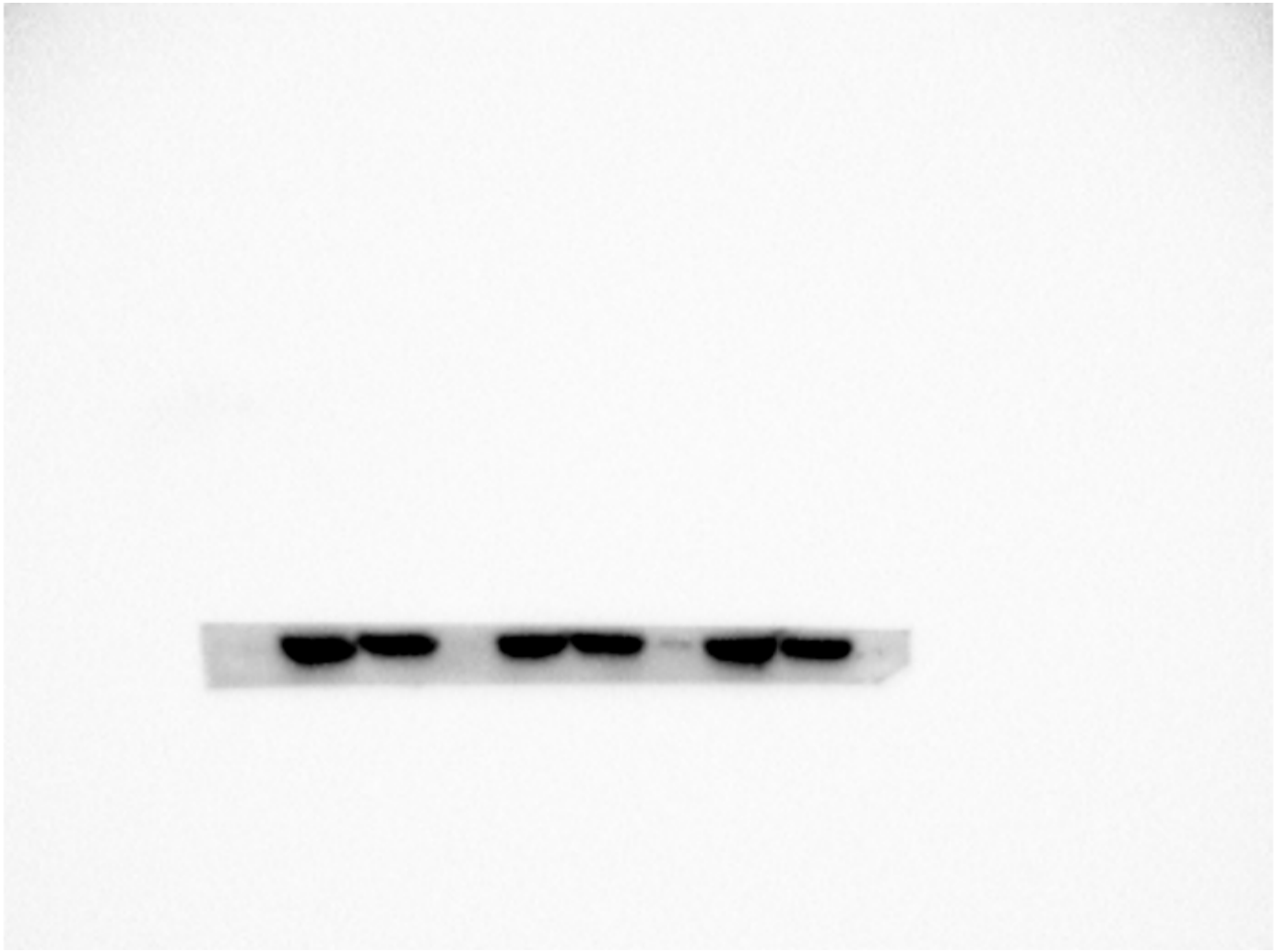

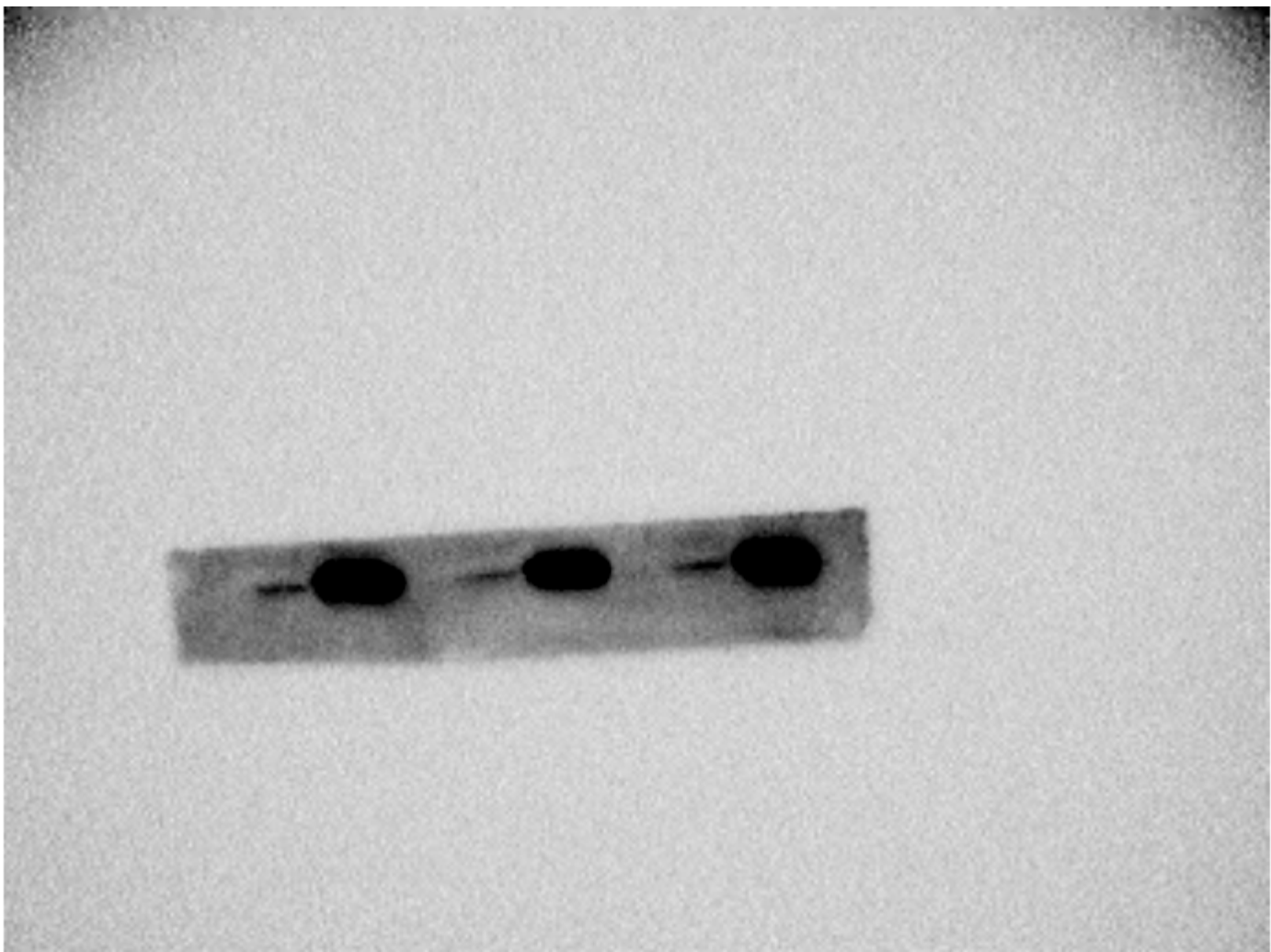

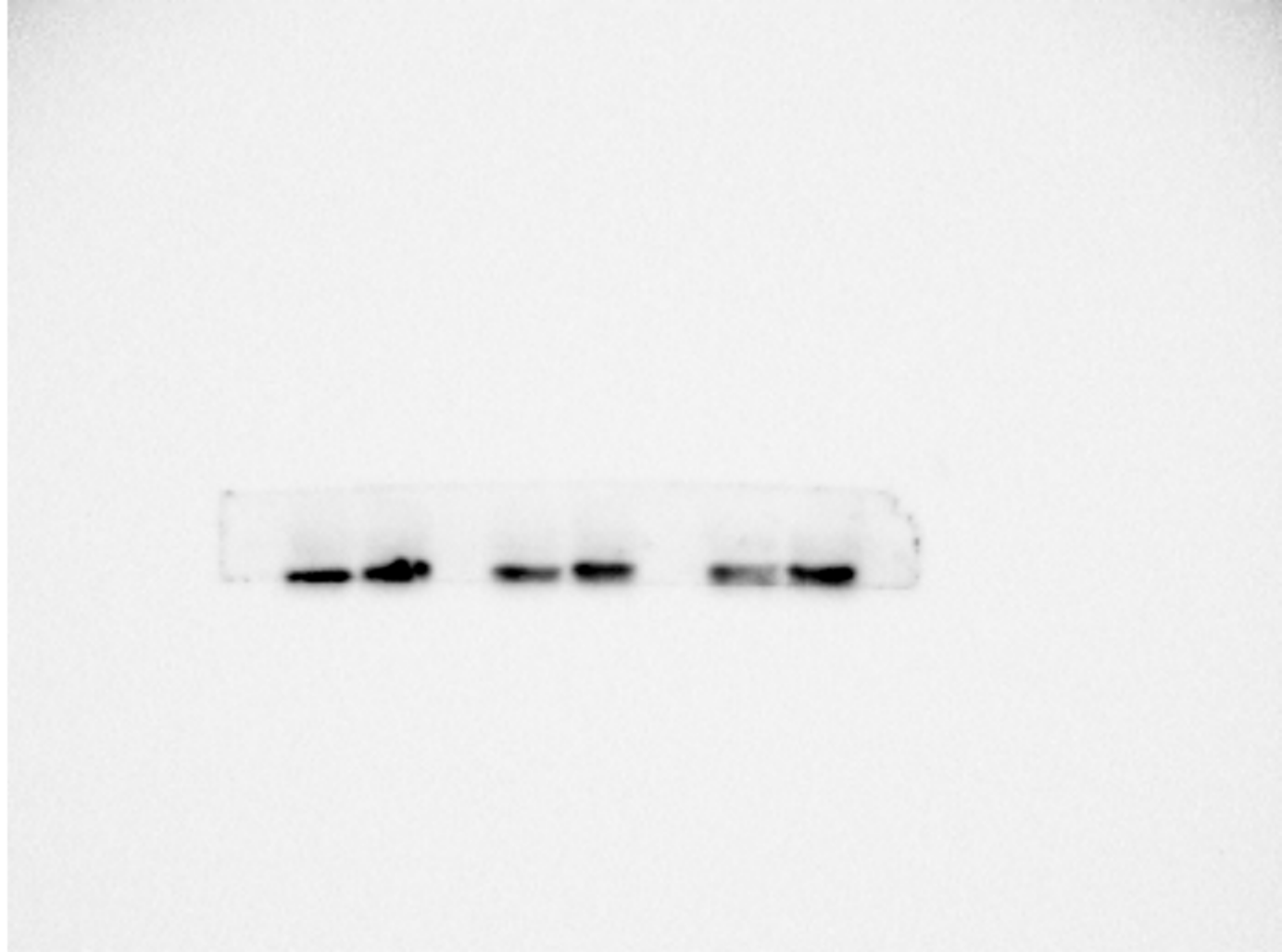

Supplement: Supplementary file 1 [file animals-14-02611-s001.zip › Western blot PDF.pdf]
