# Supplementary material for: Expression of GLOD4 in the Testis of the Qianbei Ma Goat and Its Effect on Leydig Cells
Source: Animals (Basel). 2024 Sep 8;14(17):2611. doi: 10.3390/ani14172611 (PMC11393997; doi:10.3390/ani14172611)

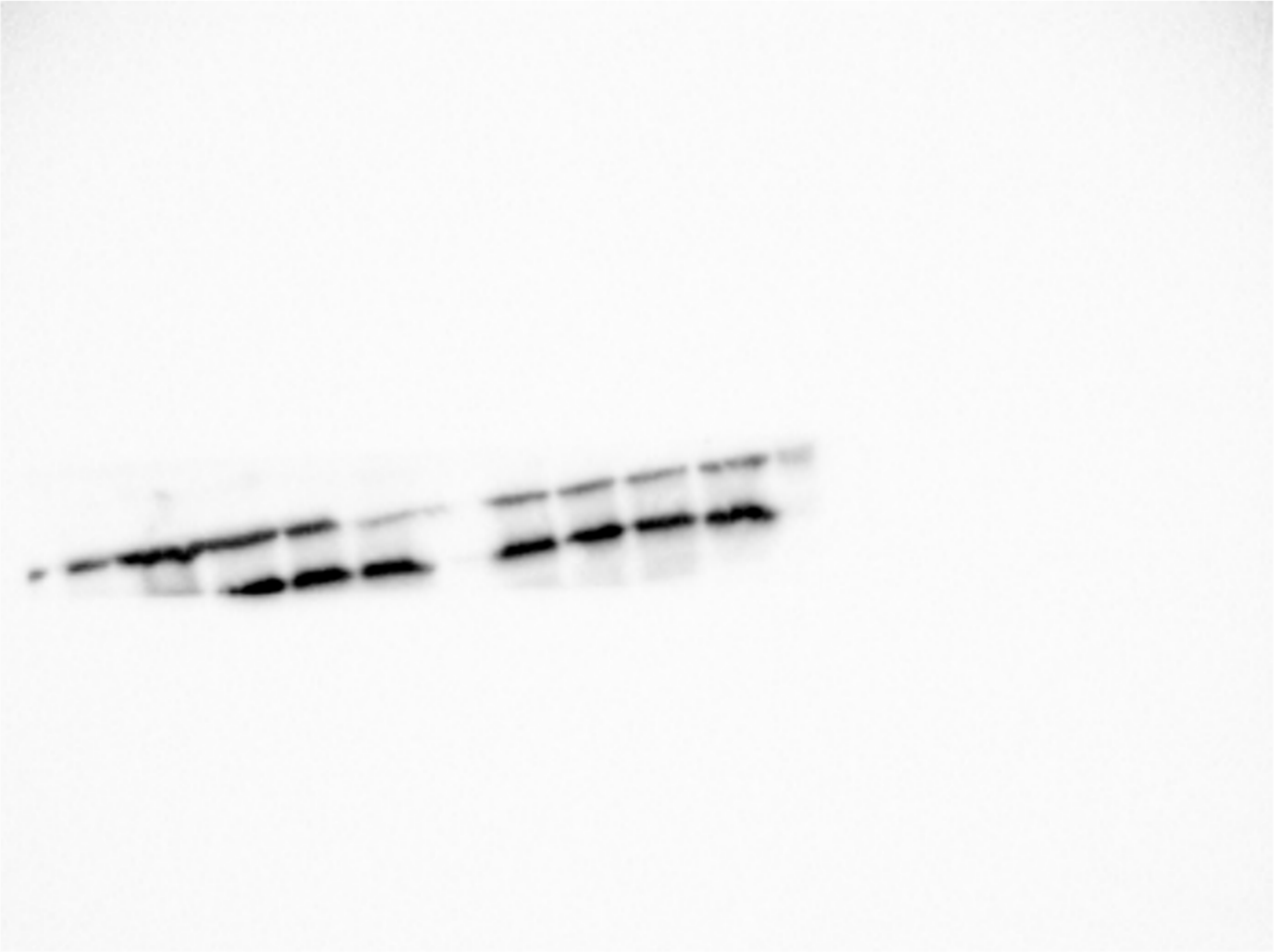

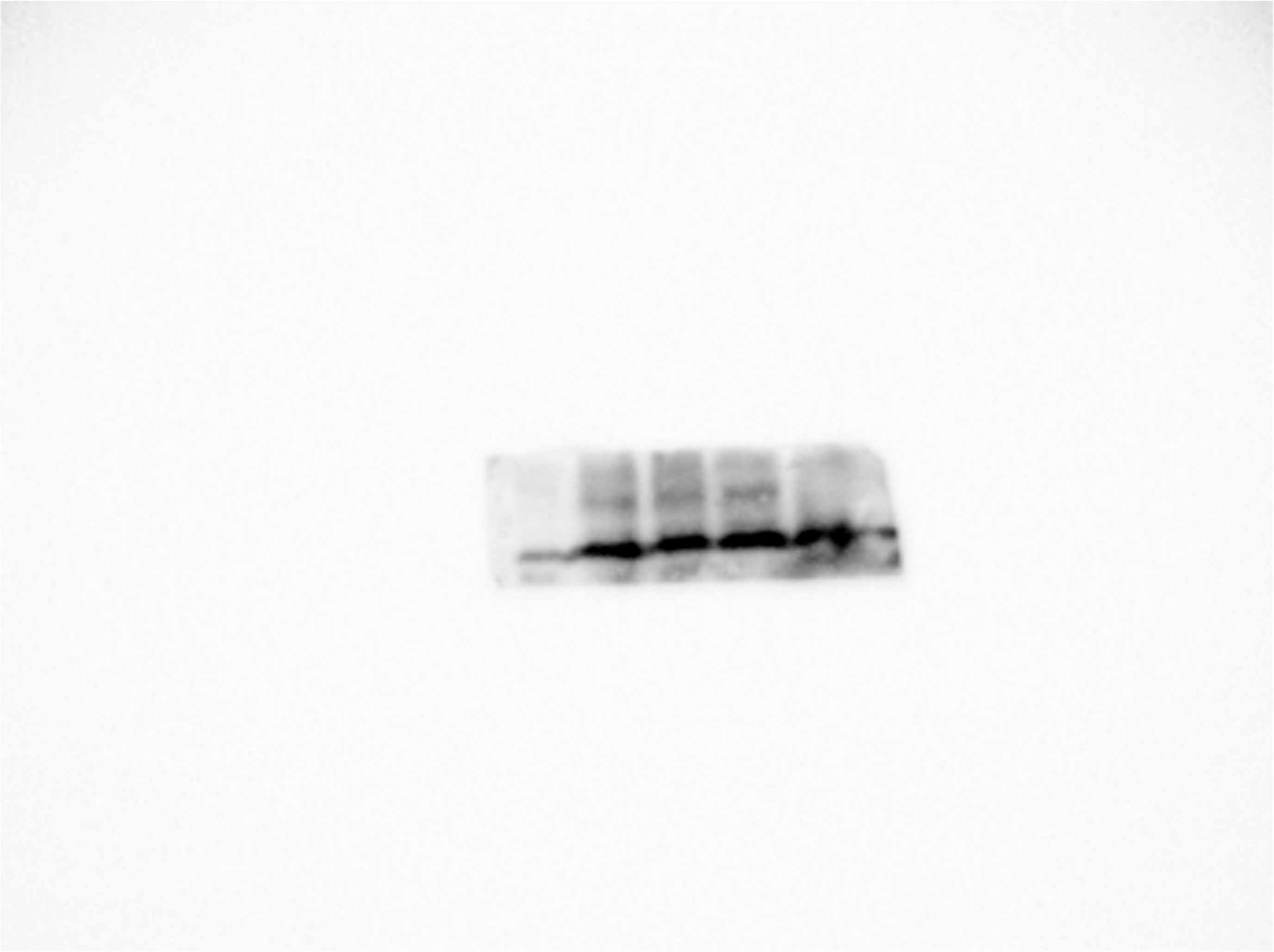

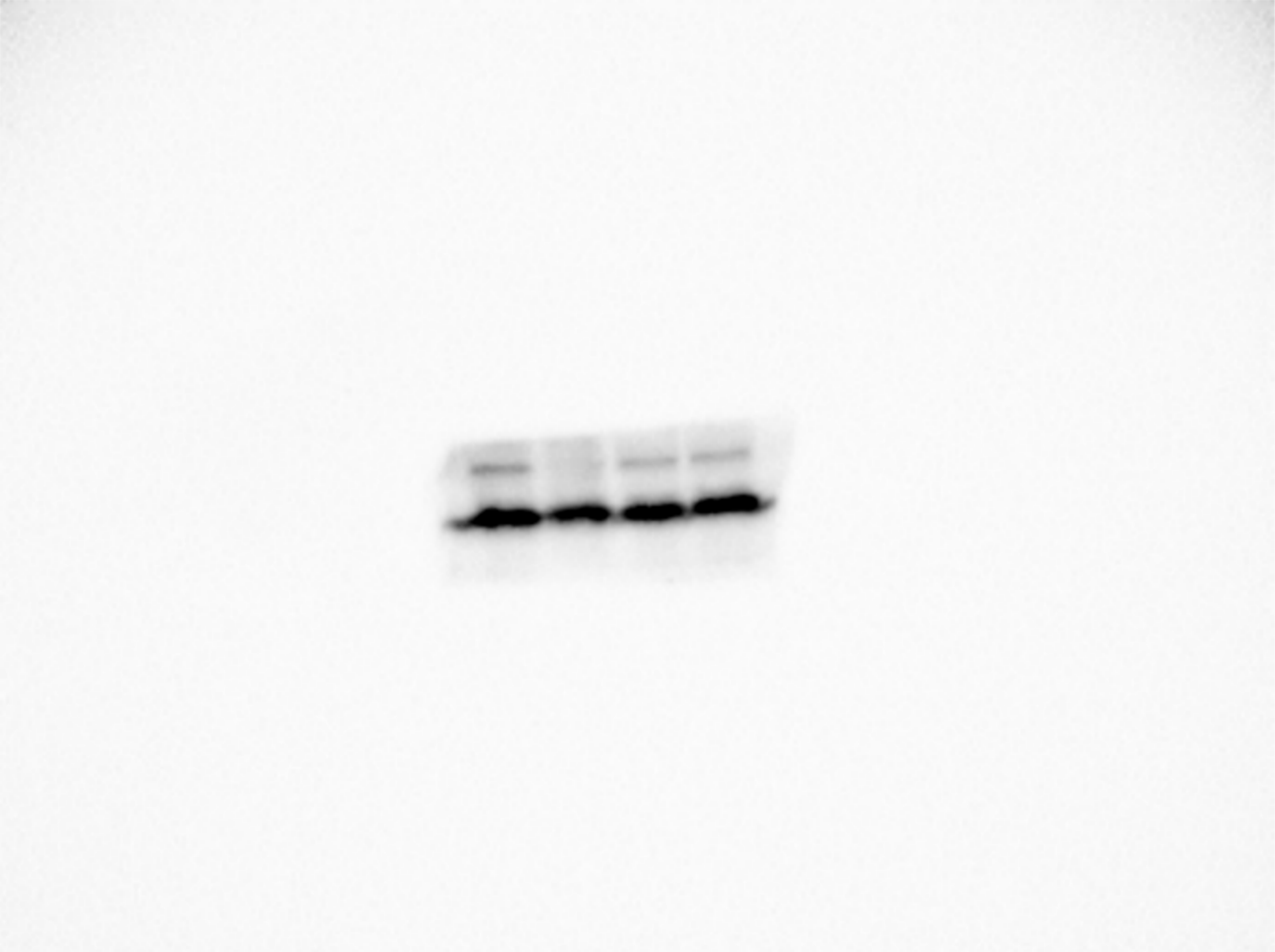

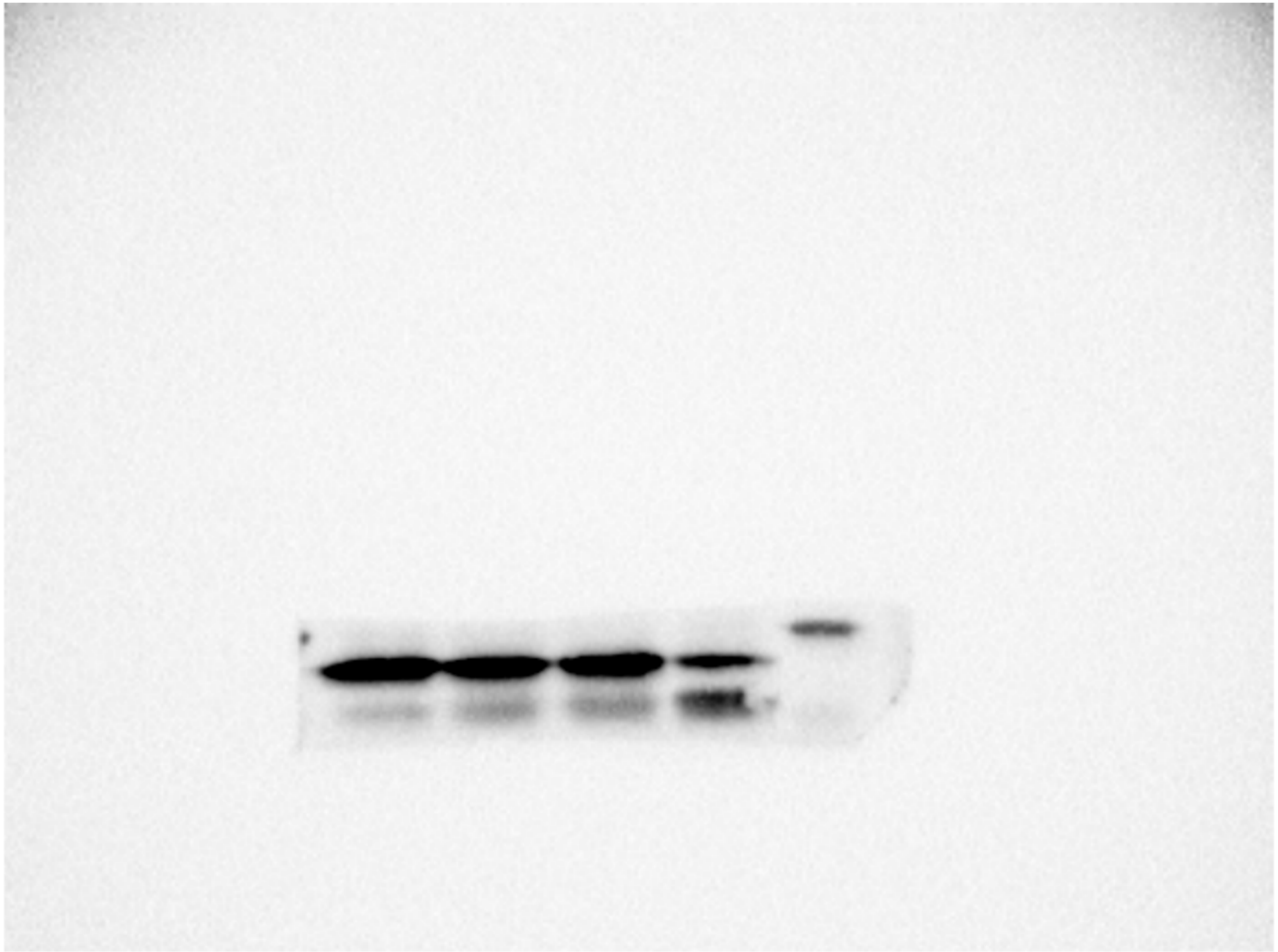

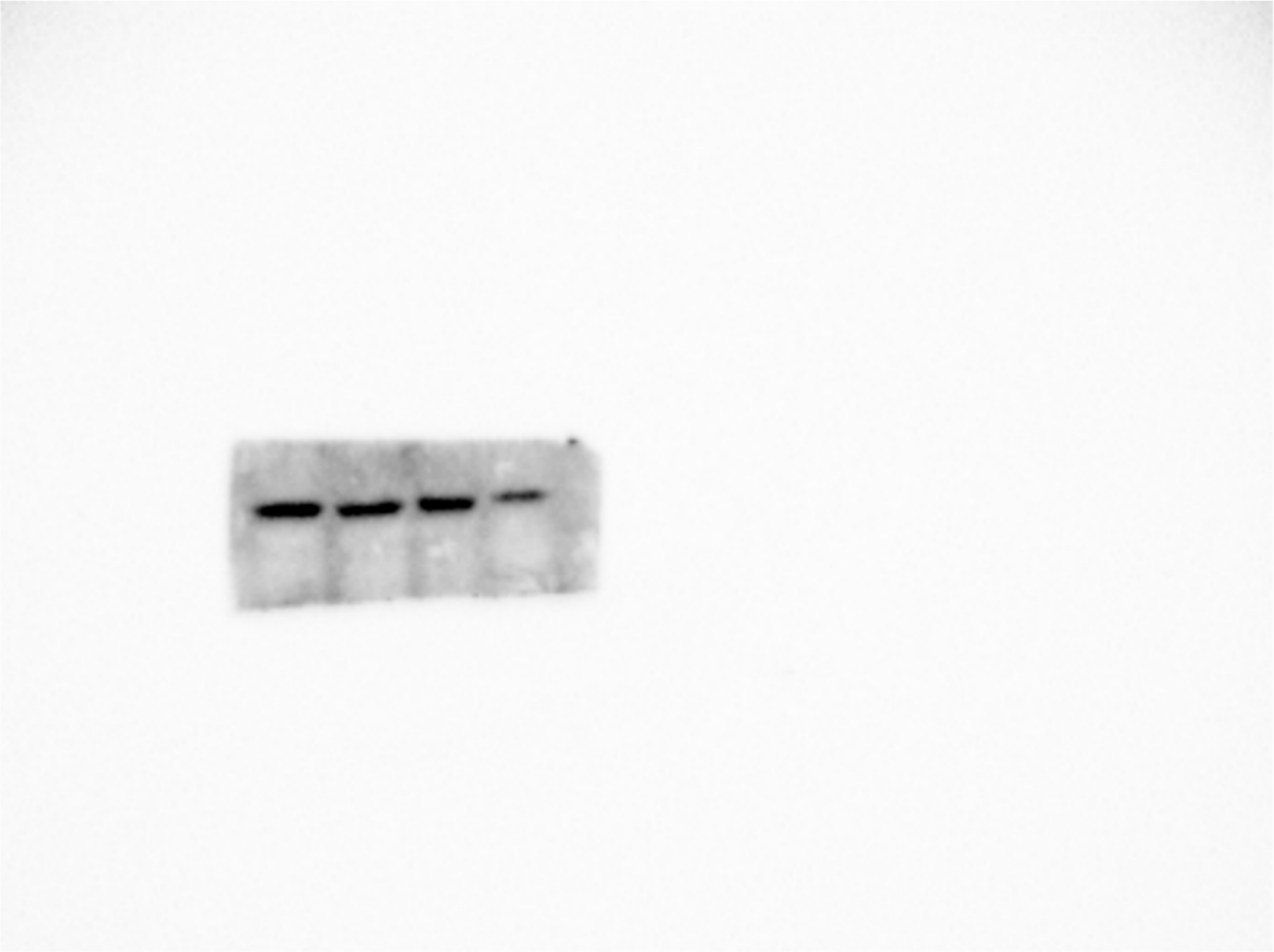

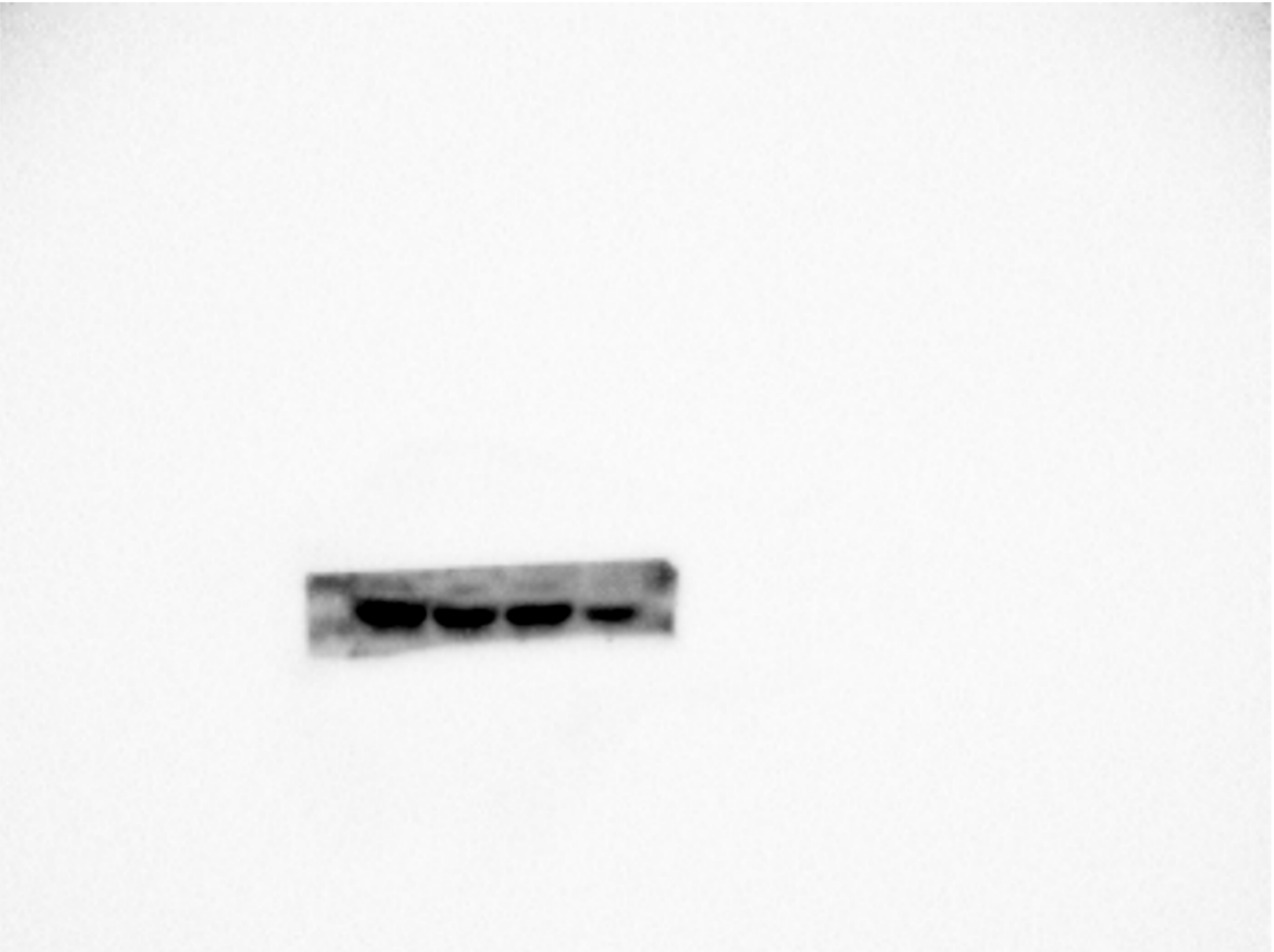

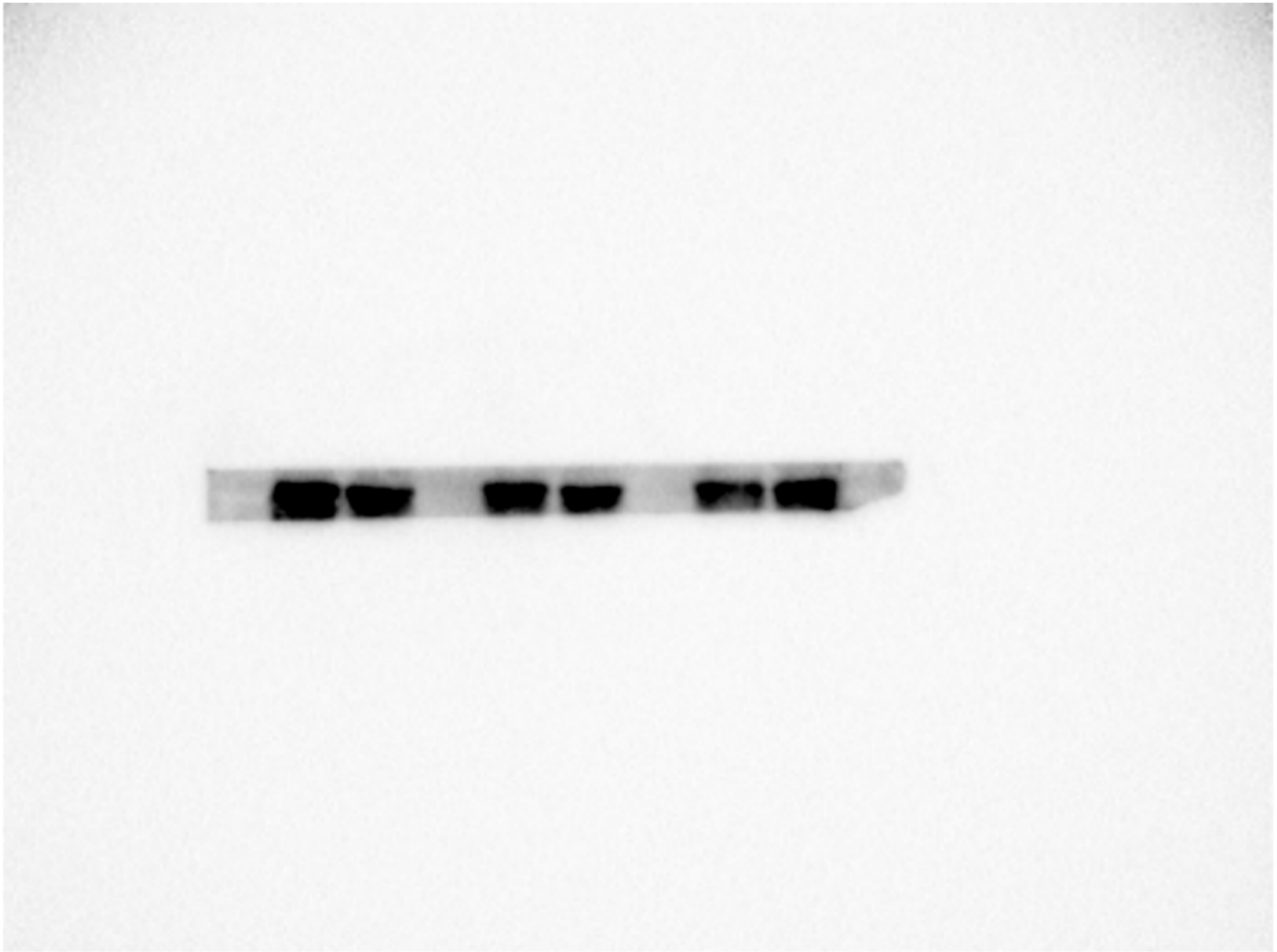

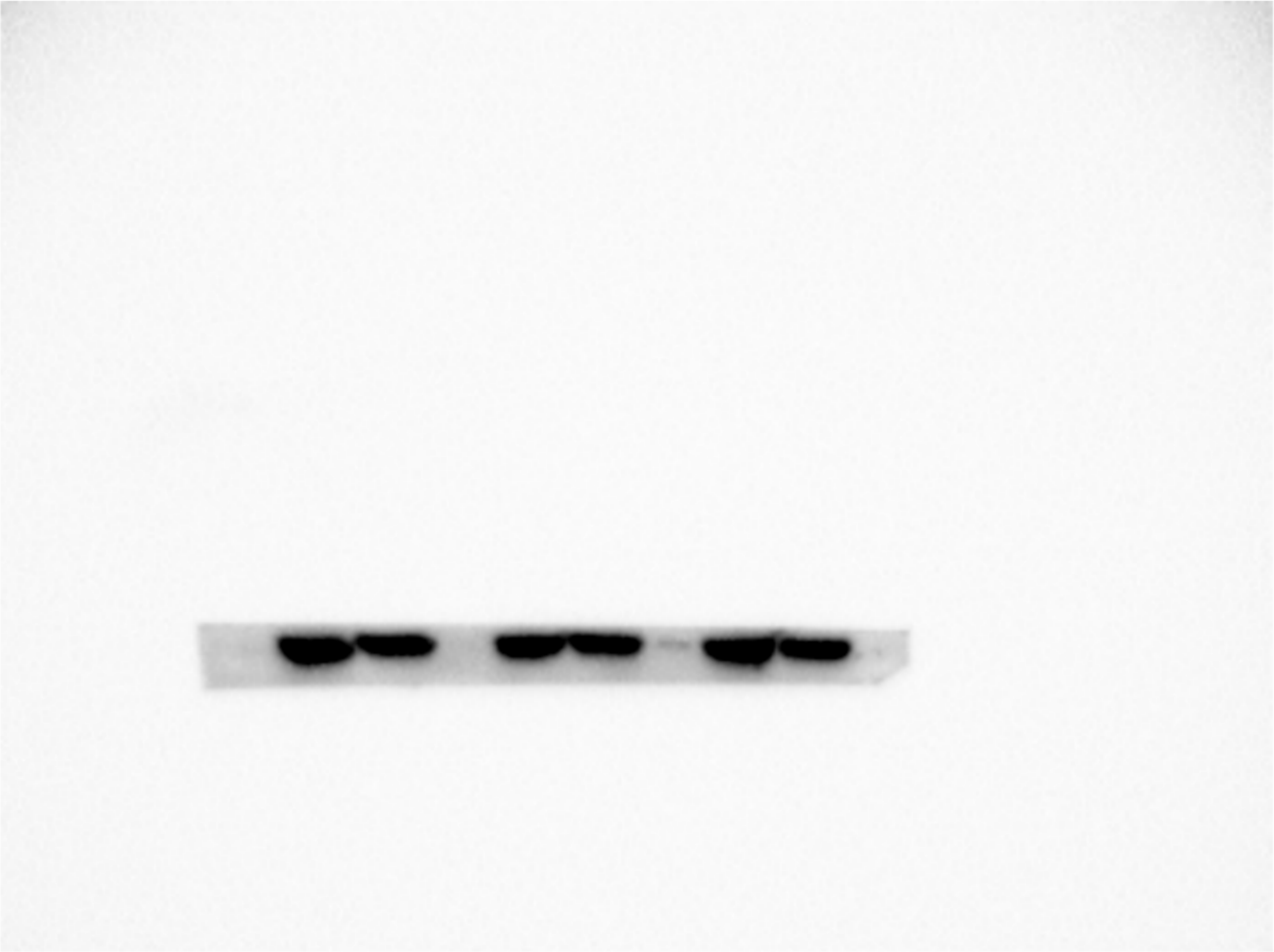

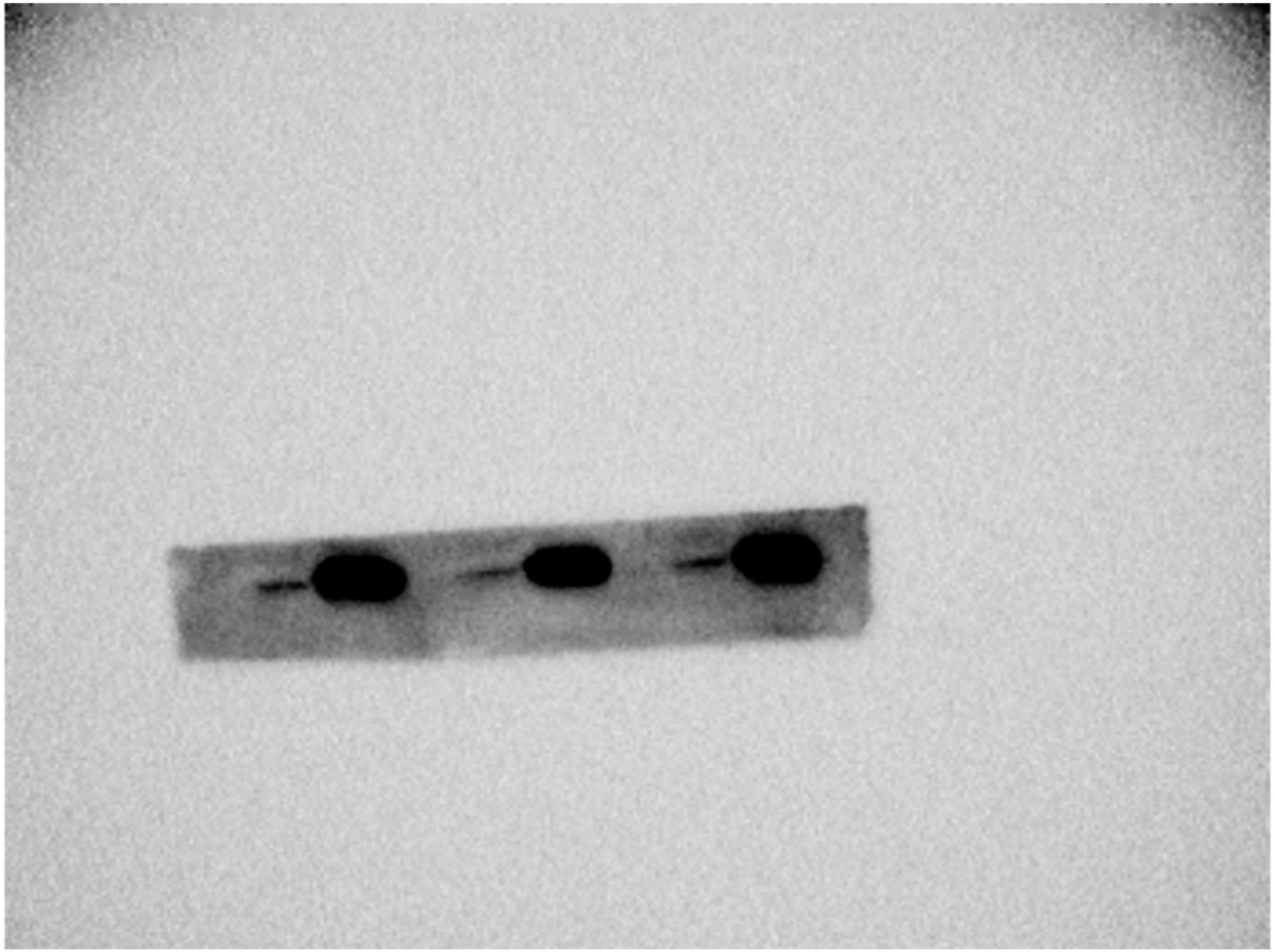

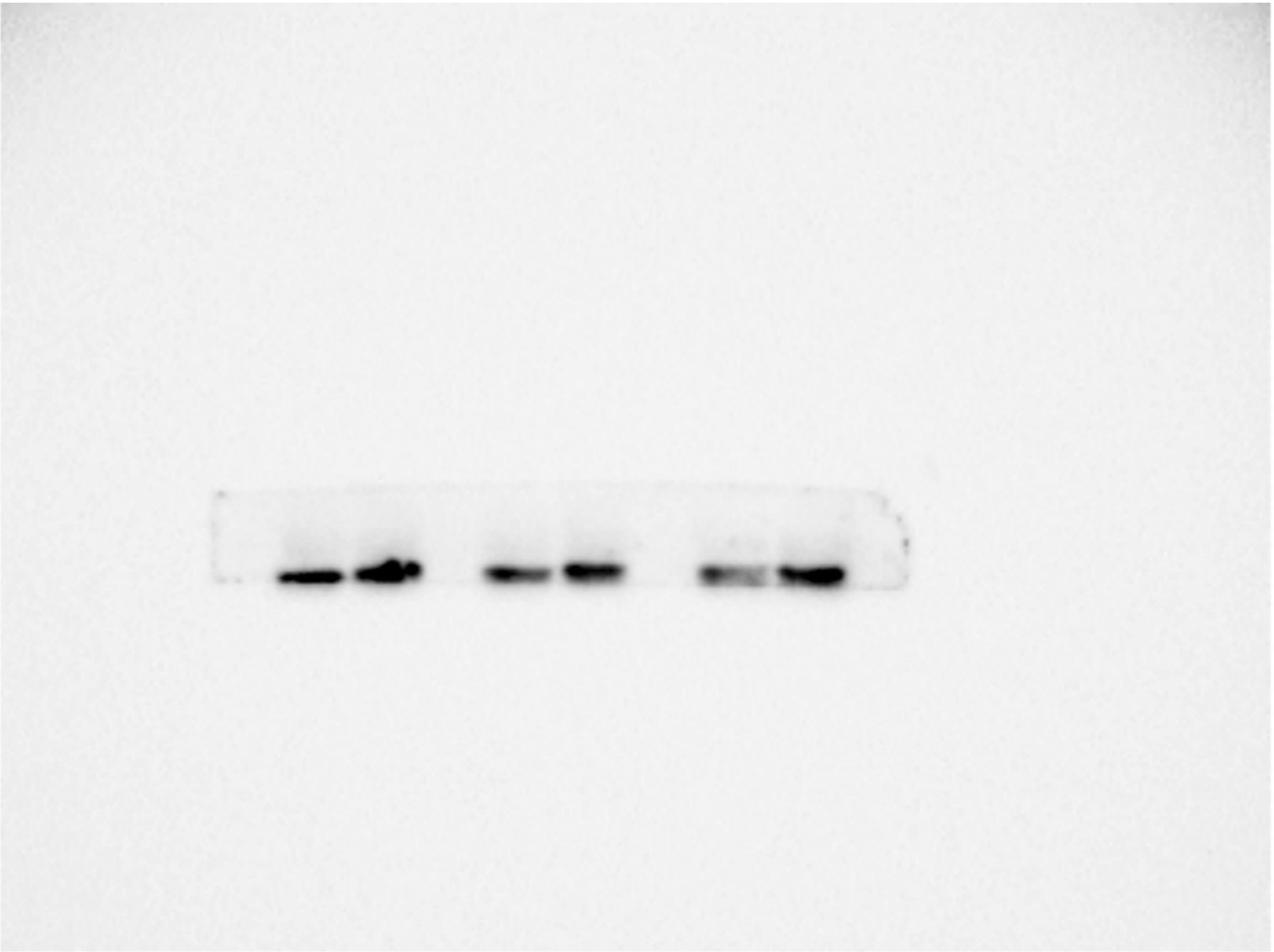

Supplement: Supplementary file 1 [file animals-14-02611-s001.zip › Western-blot images(1).zip!/Western-blot images/Western blot PDF_.pdf]
